# DnaA and the timing of chromosome replication in *Es-cherichia coli *as a function of growth rate

**DOI:** 10.1186/1752-0509-5-201

**Published:** 2011-12-21

**Authors:** Matthew AA Grant, Chiara Saggioro, Ulisse Ferrari, Bruno Bassetti, Bianca Sclavi, Marco Cosentino Lagomarsino

**Affiliations:** 1 BSS Group, Department of Physics, University of Cambridge, JJ Thomson Avenue, Cambridge, CB3 0HE, UK; 2 LBPA, UMR 8113 du CNRS, Ecole Normale Supérieure de Cachan, 61 Avenue du Président Wilson, 94235 CACHAN, France; 3 Dip. Fisica, Università "Sapienza", and IPCF-CNR, UOS Roma Piazzale A. Moro 2, I-00185, Rome, Italy; 4 Università degli Studi di Milano, Dip. Fisica. Via Celoria 16, 20133 Milano, Italy; 5 I.N.F.N. Milano, Italy; 6 Génophysique/Genomic Physics Group, UMR7238 CNRS "Microorganism Genomics; 7 University Pierre et Marie Curie, 15 rue de l'École de Médecine, 75006 Paris, France

## Abstract

**Background:**

In *Escherichia coli*, overlapping rounds of DNA replication allow the bacteria to double in faster times than the time required to copy the genome. The precise timing of initiation of DNA replication is determined by a regulatory circuit that depends on the binding of a critical number of ATP-bound DnaA proteins at the origin of replication, resulting in the melting of the DNA and the assembly of the replication complex. The synthesis of DnaA in the cell is controlled by a growth-rate dependent, negatively autoregulated gene found near the origin of replication. Both the regulatory and initiation activity of DnaA depend on its nucleotide bound state and its availability.

**Results:**

In order to investigate the contributions of the different regulatory processes to the timing of initiation of DNA replication at varying growth rates, we formulate a minimal quantitative model of the initiator circuit that includes the key ingredients known to regulate the activity of the DnaA protein. This model describes the average-cell oscillations in DnaA-ATP/DNA during the cell cycle, for varying growth rates. We evaluate the conditions under which this ratio attains the same threshold value at the time of initiation, independently of the growth rate.

**Conclusions:**

We find that a quantitative description of replication initiation by DnaA must rely on the dependency of the basic parameters on growth rate, in order to account for the timing of initiation of DNA replication at different cell doubling times. We isolate two main possible scenarios for this, depending on the roles of DnaA autoregulation and DnaA ATP-hydrolysis regulatory process. One possibility is that the basal rate of regulatory inactivation by ATP hydrolysis must vary with growth rate. Alternatively, some parameters defining promoter activity need to be a function of the growth rate. In either case, the basal rate of gene expression needs to increase with the growth rate, in accordance with the known characteristics of the *dnaA *promoter. Furthermore, both inactivation and autorepression reduce the amplitude of the cell-cycle oscillations of DnaA-ATP/DNA.

## Background

**Figure 1:**
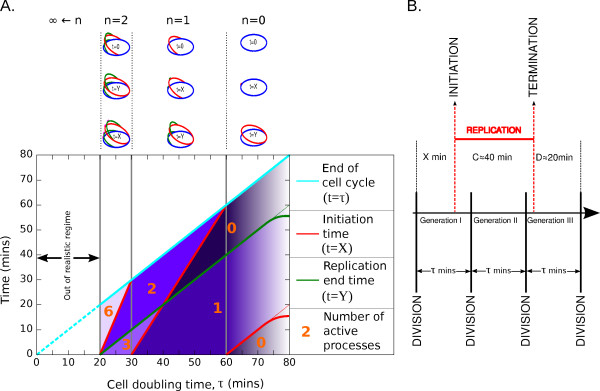
**Timing of DNA replication initiation as a function of the length of the cell cycle according to the Cooper and Helmstetter model**. A: Plots of the values of *X*, *Y *and number of active processes (termed F in the main text) in each region of the graph, for different values of the cell doubling time. The purple shading reflects the number of active processes in each region, with lighter shades denoting a greater number of active processes. Towards *τ *= 80 mins, the lines *t *= *X *and *t *= *Y *are shown curving off, showing that this is outside the regime 20 mins ≤ *τ *≤ 60 mins where the *C *and *D *periods can be considered constant. Above the graph in panel A are diagrams of the state of the chromosome for critical time values, for each of the values of *n *(the number of overlapping replication rounds). B: Illustration of overlapping replication rounds, in the case of a complete replication round of *n *= 2 overlapping rounds, and 3 generations.

The coordination of DNA replication with cell division in *E. coli *is a classic problem of bacterial physiology [1]. It is connected with the control of the bacterial DNA replication and cell division cycle as a function of the growth rate, and it is an essential component for evolutionary adaptation to fast-growing conditions [2]. It is also a classic problem for biological modeling [3-7]. The main outstanding questions have to do with the characterization of the network of regulatory interactions by which cells determine the timing of initiation and limit it to once per cell cycle. The theoretical foundations for understanding chromosome replication initiation in *E. coli *were set by Cooper and Helmstetter [8], by showing that the time taken for a single chromosome to be replicated (*C *period) and the time period between completion of chromosome replication and the following cell division (*D *period) were approximately constant for a cell doubling time of less than one hour [9]. The same work also introduced the idea of overlapping rounds of chromosome replication, where a round of replication can be initiated while an existing round of replication is still proceeding (Figure 1). This mechanism allows *E. coli *to grow with a doubling time faster than the time required to copy its genome. The question then arises of how the cell determines when to initiate DNA replication and how this is coupled to the growth rate.

In 1968, Donachie calculated that the correct timing would be guaranteed by a constant ratio of the cell size at the moment of initiation (termed the 'initiation mass') and the number of *oriC *in the cell [10]. Direct measurements of this ratio or of the initiation mass from cell population are difficult. Thus, whether this ratio is effectively a constant in cells with doubling times lower than one hour is to some extent an open question [11-14]. It was then proposed and debated that the amount of an initiating factor accumulating with the cell's mass could reach a threshold value resulting in activation of the origin(s). The DnaA protein has been shown to possess the basic characteristics necessary to act as such an initiator [15,16]. Several monomers of DnaA bind cooperatively to *oriC *and induce DNA melting required for assembly of the replication forks [17-19]. Its level of expression increases with the growth rate [20] to result approximately in a constant amount of total DnaA per cell [16]. DnaA overexpression results in earlier initiation, and when it is depleted it results in delayed initiation [21,22].

In addition, DnaA exists under two forms, ATP or ADP bound. The first is required for activation of the origin, thus it is usually called the active form [23]. After the replication of *oriC*, DnaA-ATP becomes converted to DnaA-ADP in a process known as 'RIDA' (Regulatory Inactivation of DnaA). RIDA is mediated by the Hda protein and the beta clamp subunit of the replisome and requires active replication forks [24,25]. The hydrolysis of the ATP in a DNA-replication dependent manner decreases the activity of the protein after initiation has taken place, thus reducing the probability that a new initiation event will occur within the same cell cycle [24,25]. At the same time the synthesis of new DNA creates new DnaA binding sites that can titrate DnaA from the origin [5,26,27].

Other processes can contribute to prevent reinitiation within the same cell cycle, such as the binding of the SeqA protein to the newly replicated, hemimethylated DNA [24,28,29]. It is believed that an "eclipse" period where reinitiation is not possible allows a buffer time for the other processes such as RIDA to take effect and thus for the levels of DnaA-ATP to decrease below the critical level for initiation. Several GATC sites are also found at the *dnaA *promoter but their effect on the timing of initiation remains to be established [30,31]. Finally, a set of proteins have been shown to either inhibit or enhance the activity of DnaA at the origin. These are for the most part abundant nucleoid proteins such as FIS, HU and IHF, that may play a regulatory role as a function of changes in the growth phase [32].

DnaA-ATP binding to the origin must determine the timing of initiation for a range of growth rates and thus in the presence of increasing genome amounts (providing non-specific binding sites). Thus, the amount of DnaA-ATP per cell needs to increase with the decrease in doubling time. The *dnaA *gene is found next to the origin on the chromosome, resulting in the gene copy-number increasing with the number of origins. In addition, the expression of the *dnaA *gene is growth rate-dependent [16,20,33]. The *dnaA *promoter region contains multiple binding sites for DnaA with differential affinity and specificity for the ATP- and ADP-bound forms of the protein and has been shown to be autorepressed by DnaA-ATP but not DnaA-ADP [18]. Consistent with this negative autoregulation, the artificial addition of DnaA boxes in the cell results in an increase in gene expression [27,34-36] and inhibition of the RIDA process or the presence of a mutant form of DnaA insensitive to RIDA (DnaAcos) results in a decrease in the level of DnaA protein in the cell [37,38]. Finally, DnaA is a transcription factor for a set of genes involved in regulation of DNA replication [19] and it could thus act as a reporter of the DNA replication state of the cell in order to maintain the correct stoichiometry of the DNA replication regulatory factors at varying growth rates and in response to perturbation to the movement of the replication forks [39].

It has previously been proposed that the presence of both autoregulation and RIDA contributes to increased robustness of the initiation regulatory network upon perturbations [24,37]. In this work, we aim to determine the relative roles of of these two regulatory processes in the control of the timing of initiation with changing growth rate. We begin from the elements provided by the Cooper and Helmstetter model in order to estimate the initiation time at different growth rates. The two main assumptions are that initiation of DNA replication is determined by a critical amount of DnaA-ATP per non-specific site on the genome and that this threshold value remains constant as a function of growth rate. On the other hand, the cellular and metabolic parameters can change with growth rate and have an impact on the DnaA circuit. In order to understand this, we use information from systematic studies of cellular changes with growth rate [40,41]. Finally, the volume of the cell is assumed to be a less relevant background as a reservoir of DnaA-ATP than the number of non-specific binding sites on the DNA [42].

The resulting equations describe, via a continuous change in parameter values with growth rate, the oscillations in DnaA-ATP per non-specific site and the attainment of a constant threshold as a function of growth rate. This shows that the circuit performing the timing of replication initiation must encode subtle information on the bacterial physiological state through the growth rate dependence of the parameters. This analysis also allows us to define a few scenarios consistent with the available experimental knowledge and to make testable predictions on the relative roles of DnaA autorepression and of the RIDA process at different growth rates. We use this model to elucidate the reciprocal roles of the known factors affecting DnaA activity in *E. coli*, namely that DnaA expression is dependent on transcriptional autoregulation, and that its ATP-ase activity is coupled with the activity of the advancing replication forks (RIDA). The results show that a working system can still be produced in the absence of RIDA or DnaA autoregulation. Moreover, both RIDA and autorepression contribute to a decrease in the amplitude of the cell-cycle oscillations in DnaA-ATP. RIDA has a larger effect at the faster growth rates while negative regulation has a larger effect at slower growth.

## Methods

### Assumptions of the model

The model consists of a set of Ordinary Differential Equations (ODEs) describing DnaA-ATP production by the expression of the *dnaA *gene. It is built on two basic assumptions. The first is based on the evidence that a specific number of DnaA-ATP molecules need to bind on oriC in order to create a replication bubble. Following the standard thermodynamic model of protein-DNA binding [42-44], the probability of this event is dependent on the number of DnaA-ATP molecules that are bound to the non-specific sites along the chromosome. These are low-affinity sites compared to titration sites, but the affinity is high enough so that the protein spends most of its time bound to the genome. These sequence-independent interactions are typical of DNA-binding proteins. As a consequence, the simplifying assumption is usually made [42] that the key molecular players (RNAP and TFs) are bound to the DNA either specifically or non-specifically. Simply stated, this is just an implementation of the known fact that DNA-binding proteins, besides binding tightly to their target sequences, are generally "sticky" for DNA, in a sequence-independent manner. This implies that the timing of DNA replication initiation in the cell is determined by the ratio DnaA-ATP to non-specific binding sites on DNA (which in turn must be proportional to the total DNA length of the chromosome(s) in the cell and effectively results in the computation of the amount of DNA per cell). Note that in this case the volume of the cell is not taken into consideration since it is not the change in concentration of DnaA or of DNA that determines the initiation time. The same will apply to the binding of DnaA and RNA polymerase to the *dnaA *promoter (see below). The second assumption is that at the time of initiation this ratio will be the same, independently of the growth rate and thus the number of origins.

We ask how the parameters of this model must vary in order for this assumption to hold in the range of doubling times between 20 and 60 minutes. In the absence of autoregulation, the only factor that contributes to a decrease in the ratio of DnaA-ATP to non-specific binding sites is the increase in DNA after DNA replication has begun. The complete model also includes the autoregulation of DnaA expression by DnaA binding to its own promoter [18] and DnaA-ATP transformation into DnaA-ADP through the RIDA process [37,45]. It is assumed that this ODE description is applicable to a single average-cell on time scales shorter than the length of the cell cycle. This hypothesis could be challenged for the shortest observable doubling times, but the formulation of the model is dictated by maximizing simplicity. The values of the parameters (attributed to a specific value of the growth rate) are all taken or estimated from the available experimental measurements. They are shown in Table 1, together with the sources.

**Table 1:** Initial values for the parameters in the model

Parameter	**Untransformed Value (at *τ ***= **21 mins)**	Units	Reference
Basal transcription rate *k_A_*	75	molecules/min	[70]
RNAP binding $e^{\Delta \varepsilon_{pd}∕k_{B}T}$	12/10000	Dimensionless	[42]
Dna-ATP binding $e^{\Delta \varepsilon_{ad}∕k_{B}T}$	1/10000	Dimensionless	[18], †
RNAP amount *P*_0_	5050	molecules	[41]
RIDA rate *k_R_*	10	molecules/min	[24]
Replication rate *k*_Λ_	1/40	genome equivalents/min	[8]
non-specific binding sites *N_NS_*	5 *× *10^6^	(genome equivalent)*^-1^*	[42]

The parameters are fixed with the values in the table for *τ *= 21 mins and then are able to vary for the other values of *τ *in order to fix the ratio of DnaA-ATP:chromosome length at the moment of initiation (*t *= *X*). †: Chiara Sag-gioro, Anne Olliver, Bianca Sclavi: Multiple levels of regulation in the growth rate dependence of DnaA expression, submitted.

### Formulation of the model

#### Timing of replication

We take into consideration the situation where the cell cycle repeats itself identically i.e. balanced, exponential growth. Following Cooper and Helmstetter, at a time *C *+ *D *after the initiation time, the cell divides, i.e. that time must be an integer multiple of the doubling time. Thus, if *τ *is the doubling time of the cell and *X *is the initiation time, then we must have

(1) X+C+D=(n+1)τ,

where *n *is the integer number of times that *τ *divides *C *+ *D*. *n *can be viewed as the number of overlapping rounds of replication, and 2*^n ^*is the number of origins. Thus, this equation reflects the phenomenon of overlapping replication rounds. Figure 1 shows how *X *varies with the doubling time (*τ*) of the cell. Defining *Y *as the time at which the chromosome completes replication, we have

(2) Y=τ-D.

#### Promoter term

The activity of the *dnaA *promoter is the source term for DnaA-ATP. We describe it by the standard thermodynamic model first used by Shea and Ackers [42-44]. We denote the promoter term (the number of DnaA-ATP synthesised per unit time) as *Q*, the number of non-specific binding sites on the chromosome as *N_NS _*and the number of RNA polymerase (RNAP) molecules as *P*. Furthermore, we denote the number of DnaA-ATP molecules as *A*_-_. We then use the assumption that the number of non-specific binding sites is proportional to the length of DNA in the cell (which we write as Λ), i.e. Λ = *κN_NS_*.

Thus the expression for the rate of transcription at the *dnaA *promoter can be written as (see Additional File 1, Section 2)

(3) $$Q=\frac{k_{A}\Theta }{1+c_{1}\frac{\Lambda }{P}+c_{2}\frac{A-}{P}}$$

where *k_A _*is the basal rate of transcription of the *dnaA *promoter, Θ(*t*, *τ*) is the number of *dnaA *promoters (and genes) in a given cell at a given time, and the remaining factor is the probability of RNAP binding to the promoter. The parameters *c*_1 _and *c*_2 _depend on the binding energies Δ*ε_pd _*and Δ*ε_ad _*of RNAP and *A*_- _respectively to their promoter binding sites. The binding energies are determined from the ratio of specific vs non-specific binding affinities.

(4) $$\begin{array}{ccc} c_{1} & =\frac{e^{\Delta \varepsilon_{pd}∕k_{B}T}}{\kappa } & \\ c_{2} & =e^{\left(\Delta \varepsilon_{pd}-\Delta \varepsilon_{ad}\right)∕k_{B}T}. \end{array}$$

where the exponential terms are Boltzmann weights. *c*_2 _= 0 if the promoter is not autorepressed. A version of the promoter where DnaA binding to its sites is cooperative is described in Additional File 1.

#### RIDA term

This term reflects the number of DnaA-ATP molecules that are converted to DnaA-ADP molecules per unit time by the RIDA process. As discussed in the introduction, RIDA is a process that takes place at the replication forks during DNA synthesis. We assume that the rate of conversion *k_R _*takes the same value at each replication fork. The number of pairs of replication forks at a given time, F(t), depends on which of *X *and *Y *is larger.

For *X < Y *:

(5) $$F\left(t\right)=\left\{\begin{array}{c} 2^{n}-1\;\\ 2\cdot 2^{n}-1\;\\ 2\left(2^{n}-1\right)\; \end{array}\begin{array}{c} \text{if}\;0<t<X\\ \text{if}\;X<t<Y\\ \text{if}\;Y<t<\tau \end{array}\right)$$

and for *X > Y *:

(6) $$F\left(t\right)=\left\{\begin{array}{c} 2^{n}-1\;\\ 2^{n}-2\;\\ 2\left(2^{n}-1\right)\; \end{array}\begin{array}{c} \text{if}\;0<t<Y\\ \text{if}\;Y<t<X\\ \text{if}\;X<t<\tau \end{array}\right)$$

(note that this equation is intrinsically discrete since it relates to the physical number of replication forks) and so the conversion from DnaA-ATP to DnaA-ADP takes place at a rate

(7) $$k_{R}F\left(t\right).$$

This leads to the following differential equations for DnaA-ATP (denoted *A*_-_) and DnaA-ADP (denoted *A*_+_)

(8) $$\frac{\partial A_{-}}{\partial t}=\frac{k_{A}\Theta }{1+c_{1}\frac{\Lambda }{P}+c_{2}\frac{A_{-}}{P}}-k_{R}F$$

(9) $$\frac{\partial A_{+}}{\partial t}=k_{R}F.$$

#### Term for the growth of the chromosome

The growth of the chromosome is controlled by the replication forks. Defining the rate of DNA synthesis of each pair of replication forks as *k*_Λ_, we can write

(10) $$\frac{\partial \Lambda }{\partial t}=k_{\Lambda }F.$$

Assuming that *k*_Λ _is constant, and normalizing so that Λ = 1 is the length of one full chromosome, we have *k*_Λ _= 1*/C*.

#### Main equation

**Figure 2:**
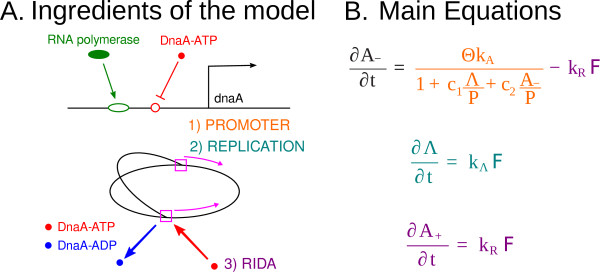
**Ingredients of the model**. A: illustration of 1) the autorepression of the *dnaA *gene 2) the growth of the chromosome by the DNA replication process 3) RIDA taking place at the replication forks. B: The key equations of the model, with the terms colour coded to match the ingredients shown in panel A. The parameters in the model are: *A*_- _(number of DnaA-ATP molecules), *A*_+ _(number of DnaA-ADP molecules), Λ (total genome length), *P *(number of RNA polymerase molecules), Θ (number of *dnaA *genes), *k_A _*(basal transcription rate of one *dnaA *gene), *c*_1_, *c*_2 _(binding constants), F (number of pairs of replication forks), *k_R _*(RIDA rate per replication fork), *k*_Λ _(growth rate of the chromosome per replication fork). The first equation represents the change in the number of DnaA-ATP molecules, with a source term due to the *dnaA *promoter (as all newly synthesised DnaA is assumed to bind to ATP due to the relative abundance of ATP in the cell), and a sink term due to the conversion of DnaA-ATP to DnaA-ADP in RIDA. The second equation represents the growth of the genome in the cell. The third equation represents the change in the number of DnaA-ADP molecules. The only source term is the same as the sink term in the DnaA-ATP equation since it is assumed DnaA-ADP is only created from DnaA-ATP during RIDA.

Figure 2 summarizes the ingredients of the model. Defining $r=\frac{A_{-}}{\Lambda }$ and combining (8) and (10) we obtain the equation

(11) $$\frac{\partial r}{\partial t}=\frac{1}{\Lambda }\left(\frac{\Theta k_{A}}{1+c_{1}\frac{\Lambda }{P}+c_{2}\frac{\Lambda }{P}r}-\left(k_{R}+rk_{\Lambda }\right)F\right).$$

This equation describes the dynamics of the variable $r=\frac{A_{-}}{\Lambda }$ which we suggest is a suitable candidate for the initiation potential since a specific number of DnaA-ATP molecules is needed to be available to bind to the origin in order to induce DNA melting, as described above. Note that usually such dynamic equations are written in terms of volume, thinking of averages over cell populations on time-scales longer than a cell cycle. We assume that the (time-varying) background of genomic binding sites is the relevant variable in a Shea-Ackers type model, extending to a single cell cycle the approach normally used for longer time scales [42-44]. We also assume that the volume can be treated as a weak perturbation, which we neglect here. In other words, the various molecules of interest (RNA Polymerase, DnaA) are partitioned between the specific and non-specific binding sites on the chromosome. Furthermore, the most important factor that determines the probability of binding to a given promoter is the absolute number of protein molecules relative to the absolute number of these binding sites, rather than the amount of protein per cell volume, which in comparison does not change significantly, and it can thus be neglected to a good approximation. Thus, this assumption means that one need not track the volume of the cell, only the number of non-specific binding sites in the cell at a given time. This idea is discussed further in Additional File 1, Section 2. Here we assume that the initiation potential, *r*, always reaches the same value at *t *= *X *independently of the growth rate and we ask how the parameters of this model must vary in order for this assumption to hold in the range of doubling times between 20 and 60 minutes. In the following, we will first establish that such a constant threshold cannot be obtained by a model with fixed parameters and then study the possible scenarios where different subsets of parameters are allowed to vary.

#### Main Assumptions

The model relies on the following further assumptions [24]: (i) All newly synthesised DnaA is immediately bound to ATP, due to the relative abundance of ATP in the cell compared to ADP and the high affinity of DnaA for ATP. (ii) DnaA-ADP is only created by conversion from DnaA-ATP by the RIDA process, when it is present. (iii) The probability of DnaA being bound to its sites at the origin or on the promoter, in the case of the presence of autorepression, is given by its thermodynamic equilibrium value. The same assumption holds for the binding of RNA polymerase to a particular promoter. This means that we assume that the rate for transcription initiation is much slower than the rates for RNAP binding and unbinding from the promoter. (iv) The rate of *dnaA *gene expression is proportional to the equilibrium probability that RNAP is bound to the *dnaA *promoter. (v) We do not consider translation directly and thus there is no time delay from transcription to protein production since the addition of this feature did not affect the result of the model (see Results). (vi) The number of non-specific binding sites on the DNA in each cell for both RNAP and DnaA is proportional to the total length of DNA in the cell [46]. (vii) The number of RNAP molecules in the cell, *P*, grows exponentially from cell birth to cell division, corresponding to the hypothesis of constant concentration and exponentially growing cell size [47]. A linear growth can also be used, however the dynamics of the model do not differ significantly between these two cases.

#### Numerical integration

The non-linearity of the main equation (11) necessitates the use of a numerical method of integration. We used a custom C++ implementation of the fourth-order Runge-Kutta method. The equation was integrated for values of the cell doubling time, *τ*, in the range 21 mins ≤ *τ *≤ 60 mins.

In order to test for the constant threshold condition, a transformation was performed by integrating the equation for *τ *= 21 mins and using the value of *r *at initiation *t *= *X *as the imposed threshold for the other values of the doubling time. Thus it was important to estimate, to a good degree of accuracy, values for the parameters at a doubling time of 21 mins. These values appear in Table 1. The parameter values are either taken to be constant (independent of cell doubling time) or are allowed to change and obtained as a consequence of the transformation (see Table 2 for whether a parameter is constant or allowed to vary in a given situation).

**Table 2:** The dependence of the parameters in the different scenarios

Scenario	Floating Parameters	Fixed parameters
1a	*k_A_*, *c*_1_, *c*_2_	*P*, *k_R_*
1b	*k_A_*, *c*_2_, *P*	*c*_1_, *k_R_*
2	*k_R_*, *k_A_*, *P*	*c*_2_, *c*_1_

In all the scenarios, *k_A _*varies with growth rate. In scenario 1a, *P *changes as a function of growth rate with the values obtained from ref. [41]. The dependence of the other parameters on growth rate is scenario specific (see Additional File 1, Figure A1).

## Results

### A fixed set of parameters gives a varying initiation threshold with increasing growth rate

**Figure 3:**
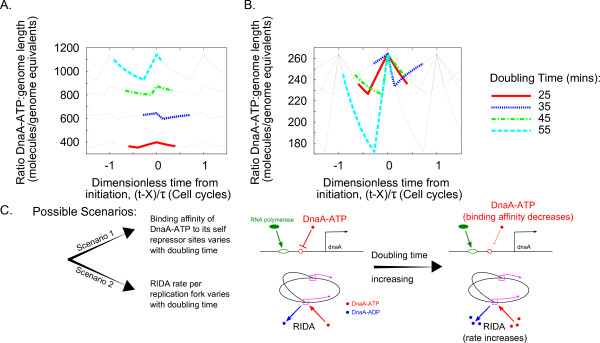
**The model imposes a specific DnaA-ATP threshold at the moment of initiation (*t *= *X*)**. A: The model with fixed parameters cannot explain an 'initiation threshold' since a different value of the ratio DnaA-ATP:genome length (*r*) is obtained at initiation (*t *= *X*) for each value of the cell doubling time, *τ*. B: We perform a mathematical transformation upon the model to impose a threshold for the ratio DnaA-ATP:genome length (*r*) at the moment of initiation (*t *= *X*) (in this specific example the threshold has been imposed by allowing the binding affinity of DnaA-ATP to its self repressor site to vary). In both panels A and B, the *x*-axis has been translated and normalized to denote fractions of cell cycles with the initiation time given by $\frac{t-X}{\tau }=0$. C: In the case in which both autorepression and RIDA are included in the model there are two scenarios in which an 'initiation threshold' can be imposed upon the model. The first of these requires the binding affinity of DnaA-ATP to its self repressor sites to decrease with increasing cell doubling time. The second scenario requires that the RIDA rate increases with increasing cell doubling time. In all scenarios the value of *k_A _*increases with increasing growth rate.

We first describe the behaviour of the model with a fixed parameter set. The ratio $r=\frac{A_{-}}{\Lambda }$ increases from the time of birth of the new cell. This can be interpreted as the accumulation of the 'initiation potential'. At initiation (*t *= *X*), *r *peaks (at the 'initiation potential' threshold) and then falls again due to the increase in the number of non-specific binding sites. When including the RIDA process, the total RIDA rate also increases following initiation, due to the higher number of active replication forks, contributing to the decrease in the initiation potential. However, the value of *r *at *t *= *X *varies for the different values of the doubling time *t *(Figure 3A). Thus, it appears that this model, with fixed parameters, cannot give a constant threshold that is reached at initiation in the range of growth rates considered here.

This fact naturally leads to us consider a model in which the parameters are able to vary with growth rate. Biologically, this is a natural requirement, as one may well expect from previous observations of the change in cellular components as a function of growth rate [40,41,48,49].

### A constant threshold condition implies alternative scenarios of growth-rate dependency in the circuit architecture

**Figure 4:**
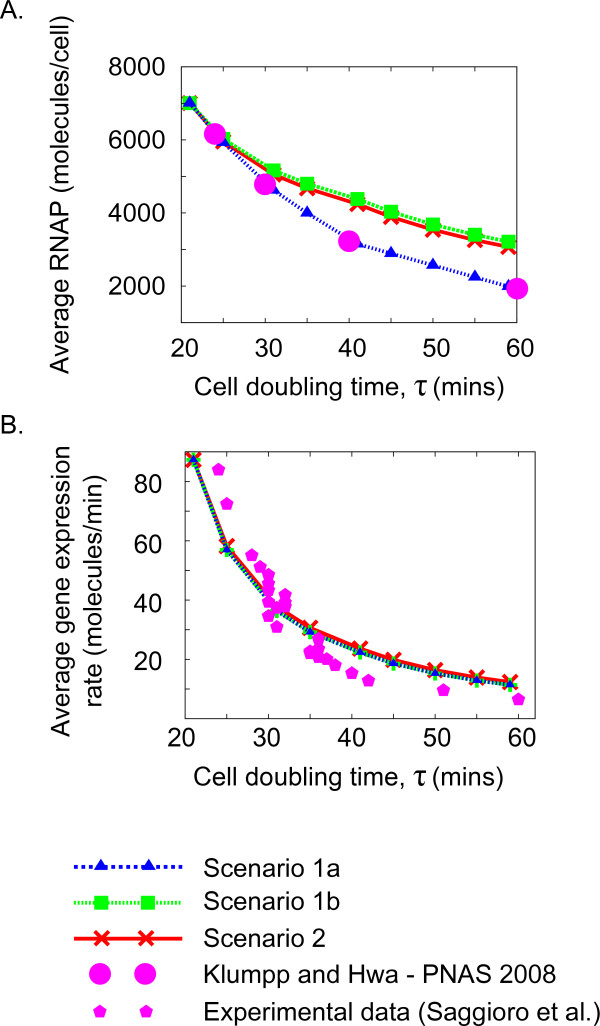
**All scenarios of the model are compatible with previous measurements and predictions**. A: Variation in the average number of RNAP molecules per cell with doubling time. Simulations from the three scenarios (connected triangles, squares and crosses) are compared to the (validated) predictions of ref. [41]. Scenarios 1b and 2 are compatible with the results (which are assumed in scenario 1a). B: Variation in the average expression rate per cell of the *dnaA *gene with growth rate in the three scenarios (connected triangles, squares and crosses) agrees with our direct measurements (Chiara Saggioro, Anne Olliver, Bianca Sclavi: Multiple levels of regulation in the growth rate dependence of DnaA expression, submitted). The measurements (pentagons) are obtained with GFP reporters on a plasmid, normalized by plasmid number and gene copy-number with varying growth rate. The experiment details are available in Additional File 1, Section 7. This prediction is also compatible with the results of Chiaramello and Zyskind [20].

**Figure 5:**
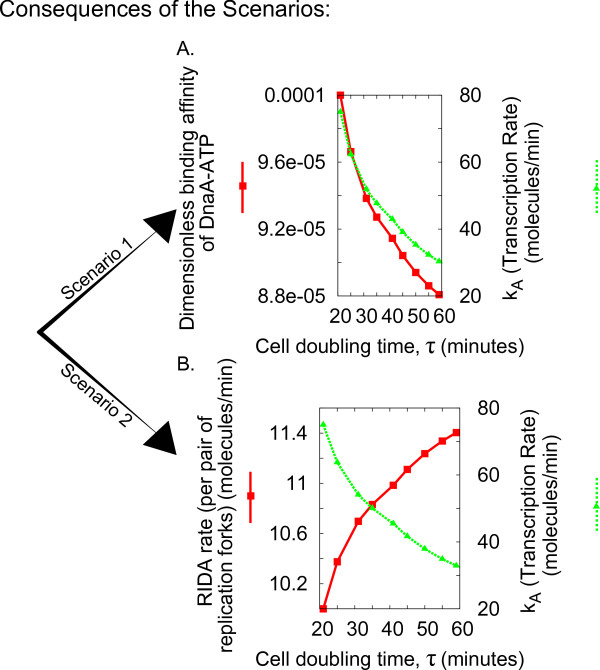
**Predictions of the model can distinguish between different scenarios**. Two possible scenarios can result in a constant initiation threshold for the model. In the first the binding affinity of DnaA-ATP to its repressor sites decreases with increasing cell doubling time, and in the second the RIDA rate increases with cell doubling time. In both scenarios *k_A _*(the basal transcription rate) must decrease with increasing cell doubling time.

The condition of a constant DnaA-ATP/DNA threshold at the time of initiation can be imposed by performing a mathematical transformation on the model and verifying the implications of this for the values of the parameters. The mathematical details of this transformation can be found in Additional File 1, Section 3. The transformation yields a fixed threshold in *r *that is reached at initiation. This can be seen upon comparing the plots in Figure 3A and 3B. In the latter, the value of *r*(*X*) is now the same at initiation for every cell doubling time. We estimate all initial values of the parameters from the literature (Table 1) and then we allow some of the parameters to change during the transformation. Thus, the transformation procedure imposes a decision on which parameters to fix and which to allow to change. This determines different scenarios, as illustrated in Figures 4 and 5 and also summarised in Table 2 (see also Additional File 1, Section 3 and Additional File 1, Figure A1). These plots explicitly show the required parameter changes at varying growth rates.

1. In the first scenario, the RIDA rate (per replication fork), *k_R_*, is chosen to remain independent of growth rate, and the other parameters are allowed to change. The transformation fixes the scaling of *k_A _*with growth rate, and provides an equation that *c*_1 _and *P *must satisfy. Since the constraints reduce to one equation with two unknowns, there is a set of possible solutions for *c*_1 _and *P*, and the scenario is underconstrained (see Additional File 1, Section 3). One possibility is to fix one of the parameters to a particular trend, in turn determining the second parameter, determining different subscenarios (Additional File 1, Figure A1).

(a) In the first of these sub-scenarios, 1a, the variation in the number of non-specifically bound RNAP in the cell, *P*, with growth rate is fixed *a priori *by imposing the trend of a previous study [41], which partitions RNAP into different classes, the RNAP bound on promoters and non-specifically bound, and determines the dependence of this partition with growth rate, (see Figure 4A). Imposing this trend determines the values for the binding affinity of RNAP to the promoter, which must vary with growth rate (Additional File 1, Section 3).

(b) In sub-scenario 1b, the binding affinity of RNAP to the *dnaA *promoter is chosen to remain independent of growth rate. Fixing this parameter constrains the remaining equation, thereby imposing a particular dependence on growth rate of the number of non-specifically bound RNAP, *P*, which turns out to be compatible with ref. [41] (see Additional File 1, Section 3 and Figure 4A).

2. In scenario 2, the binding affinity of both RNAP to the *dnaA *promoter and of DnaA-ATP to its repression sites are chosen to remain independent of growth rate (in simulations, at the values shown in Table 1), while the basal rate of transcription from the *dnaA *promoter, *k_A_*, the levels of free RNAP, *P*, and the RIDA rate (per replication fork), *k_R _*are all permitted to vary from their original values (see Figures 4A and 5B). This scenario is also feasible in absence of autorepression.

In brief, two possible scenarios can result in a constant initiation threshold for the model. In the first, the binding affinity of DnaA-ATP to its repressor sites decreases with increasing cell doubling time and in the second the RIDA rate increases with cell doubling time. In both scenarios *k_A _*(the basal transcription rate) must decrease with increasing cell doubling time. It is important to note that *k_A_*, the basal *dnaA *transcription rate, needs to vary in all scenarios with growth rate. This term (and hence its variation) is independent from RNAP availability (our *P *term) and binding (our *c*_1 _and *c*_2 _terms). It describes how quickly RNAP moves through a gene when transcribing. The variation of this characteristic time with growth rate can be associated with variations in DNA supercoling (see Discussion).

In absence of RIDA (*k_R _*= 0) or autorepression (*c*_2 _= 0), the transformation can still be performed. However, it implies that the ratio *c*_1_*/P *should remain constant with growth rate. Since *P *varies [41], this means that *c*_1 _(which, for example, could also vary through changes in supercoiling) would have to compensate exactly for the changes in *P *in order to keep a constant threshold. We have considered these further scenarios to be less probable, because they may result in a less robust control due to an unlikely fine-tuning of two parameters.

### The resulting scenarios are compatible with available knowledge on RNAP availability and total DnaA expression

Given these scenarios, we have asked whether the predicted parameter variation with growth rate and the observables quantities produced by the model were compatible with the measurements and observations available in the literature. Starting from the dependency of available RNAP with growth rate, this is predicted and matched with available experimental data in ref. [41]. Scenario 1a assumes this dependency, and therefore automatically accounts for this observation. Figure 4A demonstrates how also scenarios 1b and 2 are broadly compatible with the results of Klumpp and Hwa [41] since the average levels of non-specifically bound RNA polymerase decrease with increasing doubling time in all cases.

We now turn to the changes in measured expression of DnaA (averaged over a population) with growth rate. This can be measured by a reporter gene technique. Figure 4B shows how the model predicts that the average expression of the *dnaA *gene should change with growth rate in all scenarios. This appears to be independent of the scenario chosen and is compatible with previous findings [20]. We have also performed our own measurements, using a GFP reporter of the *dnaA *promoter encoded on a plasmid (and normalizing the result for plasmid and gene copy number (Chiara Saggioro, Anne Olliver, Bianca Sclavi: Multiple levels of regulation in the growth rate dependence of DnaA expression, submitted), see also Additional File 1, Section 7), confirming this agreement (Figure 4B).

Finally, at fixed growth rate, in order to determine whether this model reflects the main features of the regulatory network in the cell, we reproduced some of the experimental perturbations described in the literature. One of these experiments changed the rate of RIDA by changing the level of expression of the gene encoding for the Hda protein [37], while others controlled the expression of DnaA independently of the *dnaA *promoter. We found that a constant threshold can be obtained upon a 10 fold change in the RIDA rate (Additional File 1, Figure A4). In order to determine how RIDA rate and autorepression strength influence the activity of DnaA-ATP within a cell cycle in the model, we have monitored the amplitude of the oscillations of the ratio DnaA-ATP:total genome length. The results show that increased autorepression contributes to a smaller amplitude of DnaA-ATP within the cell cycle (Additional File 1, Figure A2). A decrease in RIDA has the opposite effect, and is compensated for by an increase in autorepression. In either case the inclusion of these additional control factors appears to result in smaller amplitude in the oscillation of DnaA-ATP and a smaller variation in the average amount of DnaA-ATP per cell as the growth rate is varied. This may be advantageous for the use of DnaA as a transcription factor whose activity is responsive to changes in the replication status of the cell, via the RIDA and titration effects.

Finally, once we obtained a set of parameters that satisfies the constant threshold constraint, we modified the RIDA rate while leaving the other parameters unchanged (Additional File 1, Figure A7), attempting to reproduce the effect of under or over expressing the Hda protein, as in the experiments by Riber *et al*. [37]. In our model, this results in a change of the threshold value as a function of growth rate, and more significantly at slow growth. At faster RIDA rate, the threshold value is higher at slow growth, while the opposite is observed when RIDA rate is decreased. These compatibility tests give positive results, but do not allow us to distinguish between the two scenarios. We have explored the literature for tests of dependency of the RIDA rate with growth rate, and have found no evidence of this, which lead us to consider scenario 1, where the RIDA rate is constant as the main one.

### Model Variants

In order to gain confidence that the conclusion (that the parameters need to vary with growth rate) is not a consequence of the restricted set of biological ingredients included in this model, we considered some additional model variants, including some of the known factors that may influence the timing of replication initiation.

#### 1. Delay in the synthesis of DnaA-ATP

We introduced a delay, representing the time necessary to obtain an active DnaA molecule from the binding of the RNA polymerase to the *dnaA *promoter to the end of translation. This delay, however, does not produce a significant effect in imposing a DnaA-ATP threshold at initiation, suggesting that translation delay might not have a predominant role in controlling the timing of initiation (see Additional File 1, Figure A6B).

#### 2. Cooperativity of autorepression

Cooperativity of autorepression affects the growth rate dependence of gene expression [41]. Additional File 1, Figure A6C shows by simulation that the presence of cooperativity alone cannot explain a constant threshold. Thus it is still necessary to impose a constant threshold on the model using a transformation such as that described in Additional File 1, Section 3. However, in general one cannot make a translation and scaling and keep the promoter in the same form (see also Additional File 1, Section 5), and the transformation itself poses complex constraints on the possible regulations. Thus, we decided to concentrate on the simpler non-cooperative model. Biologically, of the two high-affinity DnaA sites found at the *dnaA *promoter, one matches exactly the consensus sequence, and has a higher affinity than the other [18], suggesting that at lower DnaA concentrations only one monomer could be bound. We have verified that the transformation is possible with a promoter that would fit this profile, i.e. of the form

$$Q=\frac{k_{A}\Theta }{1+c_{1}\frac{\Lambda }{P}\left(\frac{1+\frac{r}{k_{1}}+\frac{r^{2}\omega }{k_{1}k_{2}}}{1+\frac{r}{k_{1}}}\right)},$$

where the parameter *ω *represents the cooperativity, and where *k*_1 _and *k*_2 _are the binding affinities of the two DnaA binding sites, multiplied by the proportionality constant between Λ and *N_NS_*. This description introduces two new parameters - the cooperativity and the binding affinity of the second binding site. Since these parameters appear in the equations describing the transformation that keeps the constant threshold, the scenarios become more underconstrained in a non-essential way. Thus, while a more realistic promoter model could be useful for future descriptions, we decided not to pursue it here.

#### 3. The *datA *locus and specific DnaA binding sites

We considered the effect that the presence of the *datA *locus has on the model by introducing a site on the chromosome that binds up to 300 DnaA-ATP molecules immediately after initiation has taken place (since *datA *is close to the origin on the chromosome, it is copied soon after initiation). We included one *datA *site for each origin in the cell. The results indicate that the *datA *locus might indeed prevent further initiations in a given cell cycle, since it titrates large numbers of DnaA-ATP molecules, effectively preventing them from binding at the origin (see Additional File 1, Figure A6A). However, this variant of the model fails to achieve a constant DnaA-ATP threshold at initiation at different growth rates if all parameters are kept constant. One can speculate that the large increase in *datA *sites at faster growth rates would require a proportional increase in the rate of DnaA synthesis that cannot be solely provided by the increase in gene copy number. It must be also noted that the *datA *locus appears to be unnecessary to prevent reinitiation and limit initiation of to once per cell cycle [50]. Additionally, we considered the effect of the reported ≈ 300 binding sites distributed around the chromosome [51]. These high-affinity sites would sequester DnaA in a similar way as the *datA *locus, but proportionally to the genome amount, and thus effectively decrease its concentration. This is again insufficient to guarantee a constant initiation threshold and is equivalent to rescaling the RIDA rate (see Additional File 1, Section 6.2).

#### 4. The eclipse

No constraint for the eclipse period [24,28-31] was included in this model. This proved not to be necessary, probably because there is no delay between the attainment of the threshold and the initiation of DNA replication, thus avoiding the possibility that there is an 'overshoot' of initiation potential before both RIDA and the increase of non-specific DNA can begin. On the other hand, the gene copy number immediately doubles upon initiation of DNA replication which results in a sudden increase in the number of DnaA-ATP synthesized per unit time. However, this does not result in an increase in the initiation potential due to the corresponding increase in the number of replication forks and thus on the RIDA rate and the number of non-specific titrating sites.

#### 5. DnaA-ATP Recycling regions

Genomic recycling regions catalyzing the reconversion of DnaA-ADP into DnaA-ATP [52] have been recently discovered. The quantitative contribution of this process is not clear, but within our framework it makes sense to ask how this would affect the initiation threshold, as this process is, roughly, a correction to RIDA. Precisely, assuming DnaA-ADP is not limiting, this would change the model by the substitution of *k_R _*with $k_{R}-\rho \Lambda ∕F$ where *ρ *is a fixed recycling rate. We verified that in the model this recycling term cannot by itself impose a constant threshold, while it can contribute to correcting quantitatively the effective RIDA rate (Additional File 1, Section 6.1).

Since none of these model variants qualitatively changes the behaviour of the model with respect to attaining a constant initiation threshold, they were not included in the minimal model formulation, in order to avoid confusion and proliferation of parameters. However, as shown by the variants explored above, the qualitative behaviour that the parameters of the model must vary with growth rate does not hold strictly for the minimal model only, but might be more general.

## Discussion

Standard models of bacterial regulatory circuits were adapted to situations where the growth rate is fixed [42,53]. The notion that these quantitative descriptions must account for bacterial physiology through the growth-rate dependent basic partitioning of the cell physico-chemical components is now entering the field of systems biology through a combination of new work [41,48,49,54] and reconsideration of the classics [8,40,55].

The dependency of the basic parameters on growth rate can produce notable effects on a genetic circuit, and complicates the standard descriptions [56]. In our case, the task is more difficult, as the circuit under examination is active in *determining *some features of the bacterial physiology and not only affected by them. Furthermore, on the technical level, one must produce a time-dependent description the expression of DnaA over cell-cycles of a range of durations. Perhaps also for this reason, despite the fact that the regulation of DNA replication has been a subject of intense study for over 50 years [24,57], many questions remain open. Given these obstacles, we have shown that, under a series of simplifying hypotheses, a consistent mean-field description for the DnaA/replication initation circuit is possible with varying growth rate.

Our description includes the processes that are believed to be most important for initiation of replication [24]. In these respects, it is broadly compatible with previous modelling approaches [4-7]. Its originality lies in the minimality and in the attention given to growth-rate dependency. We focused on the minimal ingredients necessary in order for the basic tenet that the ratio DnaA-ATP/DNA attains a constant threshold at initiation to hold [58,59]. The validity of this tenet is confirmed by the recent observations that initiation time is not affected by adding an extra origin on the chromosome [58] and on the compensatory mutations emerging in Hda mutants [59].

We have defined the DNA replication initiation potential, determining the (synchronous) timing of DNA replication, as the DnaA-ATP to DNA ratio, *r*. Molecular titration has been shown to result in ultrasensitive "all or none" responses [60], which further justifies using *r *as the threshold and could explain the synchrony of initiation in cells containing *oriC *minichromosomes [61]. We assume that its value at the time of initiation, *r*(*X*), is independent of the specific growth rate. The amount of DnaA-ATP at the time of initiation thus needs to increase as a function of growth rate in order for *r*(*X*) to remain constant as a function of doubling time, and we found that consequently, some of the model's parameter values must be allowed to vary. This assumption has not been verified directly. On the other hand, we feel that our point of view would still be useful in case of a growth-rate dependent *r*(*X*), as it is unlikely that this dependence would automatically match the dependence of all the other parameters.

We have defined two main scenarios in which different subsets of the parameters are allowed to change. In Scenario 1 the RIDA rate (per replication fork), *k_R_*, is held constant as a function of growth rate, but the binding affinities of RNAP and DnaA-ATP to the DNA need to vary with growth rate (note that in addition, there are two technical sub-scenarios to Scenario 1 due to the possibility of either fixing the growth rate dependence of *P*, the number of available RNA polymerase molecules a priori to the trend of ref. [41](Scenario 1a) or allowing it to be free (Scenario 1b)). In Scenario 2 the binding constants (c.f. *c*_1 _and *c*_2_) are independent of growth rate but the RIDA rate, *k_R_*, must vary. We have verified that both scenarios are consistent with the eperimentally tested predictions of RNAP availability with growth rate [41] and with previous measurements [20] and our own experimental evaluation of total DnaA expression (Chiara Saggioro, Anne Olliver, Bianca Sclavi: Multiple levels of regulation in the growth rate dependence of DnaA expression, submitted), and also with a number of "in silico mutations" inspired by the available literature [24,37]. Thus, the scenarios appear as possibilities that are testable, but for the moment remain open. Note that the property that the initiation threshold holds constant with respect to growth rate changes *is not *related to the specific set of parameters we used, or any set of parameters. Our analysis shows that in general, for any fixed parameter set at a given growth rate, a transformation is necessary in order to keep the threshold constant while moving to another growth rate. In order to provide specific examples, we have produced plots in the style of those in Figure 5, with different curves corresponding to choosing different values of the initial input parameters. These demonstrate that the qualitative behaviour of the transformation is independent of these parameters (Additional File 1, Figures A8, A9 and A10). This exercise is also important to show that the parameter changes with growth rate are not numerically negligible for empirically plausible parameters, so that the question of keeping the initiation threshold constant is not purely academic.

It is then interesting to ask which of these scenarios is more reasonable considering the known biological processes. We speculate that scenario 2 is less likely, since until now there is no evidence pointing to a possible change in the intrinsic RIDA rate as a function of growth rate. The DnaA-related protein Hda (Homologous to DnaA) mediates this process [57]. Experiments with mutants over-and underexpressing Hda [37], with corresponding increases and decreases in the RIDA rate, suggest a possible mechanism by which the *k_R _*term in the equations could vary by a growth rate-dependent expression of the Hda gene. There may also be other, as yet unknown, factors that affect the growth rate dependence of the RIDA rate. Alternatively, we can speculate that the decrease in the rate of RIDA with growth rate could be caused effectively by the action of the reverse process of DnaA-ATP recycling by the recently discovered recycling regions [52]. Figure 5b shows that the RIDA rate should increase with cell cycle time and thus decrease with growth rate. This growth rate increase causes overlapping replication rounds, and thus higher chromosome copy number. Since more recycling regions are present there is more recycling, *i.e*. a decrease in the effective RIDA rate, compatibility with the requirement imposed by our results. However, considering explicitly this model variant, we find that the balancing recycling cannot by itself impose a constant threshold.

Conversely in scenario 1, the RIDA rate per replication fork is constant, and one has to rationalize the variation of the binding affinities. It seems possible that the binding affinities could change with growth rate through changes in supercoiling, in similar ways to those seen in Figure 5 and Additional File 1, Figure A5 [62,63]. The levels of average negative supercoiling are known to increase as the growth rate increases [64]. However, it makes sense to challenge the validity of the basic assumption that the ratio of DnaA-ATP to DNA at the time of initiation is constant. This model assumes that the affinity for DnaA-ATP binding to its own promoter can change with growth rate but its affinity for the origin does not. The first assumption mainly allows the model to change the magnitude of negative autoregulation as a function of growth rate, and it may indeed be explained by the changes in global cellular parameters such as negative supercoiling. We have considered how the activation threshold in the model (estimated in Additional File 1, Figure A11 and corresponding caption) would be affected if the binding affinity for DnaA to the origin would vary in the same way as its value at the dnaA promoter, required by Scenario 1, for a set of realistic parameters. We found that these changes in *r*(*X*) are less than 10% over a wide range of growth rates, suggesting that this scenario might be robust. Indeed, the observed threshold is certainly approximately constant when compared to the untransformed case i.e. the different values of the ratio at *t *= *X *shown in Figure 3A. More generally, the initiation of DNA replication has been significantly simplified in this model; all it requires is a specific amount of available DnaA-ATP molecules. However we know that other factors, such as the binding of nucleoid proteins FIS, IHF, H-NS and HU, may contribute to the formation of an open complex at the origin. On the other hand, other recent results have shown that at slower growth (slower than the range considered here) the cell contains a greater average amount of DnaA-ATP per origin that results in initiation events that are independent of the novel synthesis of DnaA-ATP [65]. These results suggest that the regulation of the initiation process at the origin might indeed be dependent on the growth rate and that these changes still remain to be characterized quantitatively before they can be included in a theoretical model.

Interestingly, the basal rate of transcription of the *dnaA *gene, *k_A _*must vary in both scenarios. Figure 5 shows that *k_A _*decreases as the cells grow more slowly. This is what is expected from a promoter like the one of the *dnaA *gene that closely resembles ribosomal RNA promoters. This family of promoters have a GC-rich sequence at the transcription initiation site called a discriminator region. This region renders the activity of the promoter sensitive to the degree of negative supercoiling, which activates transcription by enhancing DNA melting, and leads to its inhibition by the accumulation of ppGpp at slower growth rates [66].

## Conclusions

All things considered, we can say that perhaps our main result is that the determination of the timing of initiation by DnaA, besides relying on the known "architecture" comprising autorepression, RIDA and a number of other "dedicated" processes, can be understood only in its complex interplay with bacterial physiology (comprising DNA supercoiling, ppGpp, growth-rate dependent partitioning of molecular machinery, etc.)

Nevertheless, it makes sense to ask whether this model allows us to elucidate some features of the reciprocal role of RIDA and DnaA autorepression, its two main ingredients. Biologically, RIDA renders the control of DnaA-ATP dependent upon ongoing DNA replication, and thus results in an increase in DnaA-ATP when replication forks are blocked. Autorepression however probably plays a larger role in the absence of RIDA at slow growth, or in bacteria that do not have RIDA at all (such as *B. subtilis*, where DnaA titration at the replication fork seems to play an important role) [57]. The Hda protein and thus the RIDA process seems to be quite specific to the fast-growing *E. coli *bacterium and its close relatives in the *Enterobacteriaceae *(UniProtKB), suggesting that in other bacterial species this level of regulation may not be required and is replaced instead by protein degradation, e.g. in *Caulobacter *[67,68], or a high intrinsic ATPase activity of the protein, as in *Mycobacterium tuberculosis *[69]. We have verified that the model can work in the absence of autorepression or RIDA, but the tuning of the parameters to achieve a constant threshold is more "difficult", in the sense that it requires more fine-tuning of the parameters, since the ratio *c*_1_/*P *should remain constant with growth rate. meaning that it is possible that a smaller range of growth rates would be accessible in these conditions. Moreover, in the model, increasing autorepression or RIDA rate results in a smaller amplitude of the oscillations of the ratio DnaA-ATP/DNA during the cell cycle, and in a smaller variation in the average amount of DnaA-ATP per cell as the growth rate is varied. This may be advantageous for the use of DnaA as a transcription factor which has to sense perturbations in the replication status of the cell at all growth rates.

## Competing interests

The authors declare that they have no competing interests.

## Authors' contributions

MCL, BS, and BB designed research. MG, BB, MCL, UF, and CS performed research. MG, BS and MCL wrote the paper. All authors read and approved the final manuscript.

## Supplementary Material

Additional file 1 **Additional Text and Figures**. Single pdf file containing the additional text and figures mentioned in the main text. Click here for file
