# Supplementary material for: DnaA and the timing of chromosome replication in Es-cherichia coli as a function of growth rate
Source: BMC Syst Biol. 2011 Dec 21;5:201. doi: 10.1186/1752-0509-5-201 (PMC3309966; doi:10.1186/1752-0509-5-201)
Supplement: Additional file 1 — Additional Text and Figures. Single pdf file containing the additional text and figures mentioned in the main text. [file 1752-0509-5-201-S1.PDF]

Additional Text and Figures

Section 1 Considerations on the initiation mass

The initiation mass of *E. coli*, a concept put forward by Donachie in 1968 [1], is an idea that, while independent of the work presented in this paper, has been at the centre of the research into initiation of chromosome replication since its inception. This section presents some thoughts on the implications of our model on the initiation mass argument.

As explained in the main text, steady exponential growth of the cell mass is assumed. The volume of the cell at a time t, denoted $\Omega(t)$, is thus

$$\Omega(t) = \Omega_0 e^{\alpha t}, \tag{12}$$

where α is the growth rate such that

$$\alpha = \frac{\log(2)}{\tau} \tag{13}$$

If it is assumed that the initial mass of the cell is an exponential function of the growth rate [2], it is possible, for example, to write

$$\Omega_0 = \Omega_0(\alpha) = \frac{e^{\alpha(C+D)}\Omega_*}{2}, \tag{14}$$

where Ω_* is a constant.

With (12) and (13), this implies that

$$\frac{\Omega(X)}{2^n} = constant = \Omega_*, \tag{15}$$

where 2^n is the number of *oriC* in the cell. This gives a constant ratio of *oriC* to mass at initiation and so is in agreement with what was shown by Donachie [1].

Donachie's observations of a constant initiation mass are based on the observation by Schaechter et al. [2] of the initial mass of a cell growing exponentially with growth rate. As discussed, for the ratio of the mass to number of origins to be constant at initiation, a very specific initial cell mass is required, namely

$$\Omega(0) = \frac{\Omega_*}{2} e^{\alpha(C+D)}, \tag{16}$$

if the Cooper-Helmstetter model is assumed. However, this particular trend of initial mass with growth rate is not claimed to hold by Schaechter and coworkers: it is simply an exponential relationship that is claimed to hold. Thus, if the relationship was slightly different, say, for example, $\Omega(0) = \frac{\Omega_*}{2} e^{\alpha(C+D+1)}$ then one would have

$$\frac{\Omega(X)}{2^n} = \Omega_* e^{\alpha}, \tag{17}$$

i.e. the initiation mass would also be an exponential function of the growth rate. So, under the assumption of an initial cell mass varying exponentially with growth rate, it is not necessary to have an initiation mass that is constant; an initiation mass that is a continuous function of growth rate is possible. This study is concerned with determining whether DnaA can fulfill a threshold condition at $t = X$; this question is independent of whether the origin to mass ratio is constant or not at $t = X$.

Section 2 Thermodynamic model for the promoter

This study describes promoter activity using the thermodynamic model first introduced by Shea and Ackers [3, 4]. This section introduces the mathematics of the derivation of this term. Note that the term derived here is for the simple autorepressor, which is the form used in the equation (11).

The Shea-Ackers model calculates the probability (assuming equilibrium binding) that RNAP is bound to a given promoter [5]. The validity of the equilibrium binding assumption relies on the rate of binding and unbinding of RNAP to and from the promoter being much faster than the rate of open complex formation. The same is true for the relevant transcription factors (such as DnaA-ATP in this case). Under these assumptions, the probability that RNAP is bound to a particular promoter (in the absence of transcription factors) is given by the ratio of the statistical weight of the state in which RNAP is bound to that promoter, divided by the sum of the statistical weights of all possible states. To formalize this, the number of non-specific binding sites on the chromosome is denoted as N_{NS} and the number of RNAP molecules as P. The number of ways of arranging all the RNAP molecules in the non-specific sites is:

$$\frac{N_{NS}!}{P!(N_{NS} - P)!}, \tag{18}$$

and the statistical weight of this state is thus

$$Z(P) = \underbrace{\frac{N_{NS}!}{P!(N_{NS} - P)!}}_{\text{number of arrangements}} \times \underbrace{e^{-P\varepsilon_{pd}^{NS}/k_B T}}_{\text{Boltzmann weight (binding energy)}}. \tag{19}$$

And so, the probability of having RNAP bound to a given promoter is given by

$$\mathbb{P}(\text{RNAP bound}) = \frac{Z(P-1)e^{-\varepsilon_{pd}^{S}/k_BT}}{Z_{tot}(P)} \ , \tag{20}$$

where $Z_{tot}(P)$ represents the sum of the possible statistical weights, namely

$$Z_{tot}(P) = \underbrace{Z(P)}_{\text{promoter unoccupied}} + \underbrace{Z(P-1)e^{-\varepsilon_{pd}^{S}/k_BT}}_{\text{RNAP bound to promoter}} . \tag{21}$$

What can be seen is that this form of the derivation for the probability of the RNAP being bound to a given promoter does not require the volume to be considered explicitly. The spatial distribution of RNAP within the cell is not considered as it is assumed to provide only a weak perturbation to the probability, given the large number of RNAP molecules in the cell [5].

Now consider the probability for the promoter in the both more relevant and complex case where a repressing transcription factor is involved (namely DnaA-ATP).

The notation used is as follows: Q is the promoter term; A is the number of DnaA-ATP molecules as (where the subscript used in the main text has been dropped for ease of notation); N_{NS} is the number of non-specific binding sites on the chromosome; P is the number of RNAP molecules.

The number of ways of arranging the RNAP molecules and the DnaA-ATP molecules in the non-specific binding sites is

$$\frac{N_{NS}!}{P!A!(N_{NS}-P-A)!} \tag{22}$$

which is effectively the number of arrangements where the appropriate promoter is unoccupied. The statistical weight for this can be written

$$Z(P,A) = \underbrace{\frac{N_{NS}!}{P!A!(N_{NS}-P-A)!}}_{\text{Number of arrangements}} \times \underbrace{e^{-P\varepsilon_{pd}^{NS}/k_BT}}_{\text{weight of each RNAP state}} \times \underbrace{e^{-A\varepsilon_{ad}^{NS}/k_BT}}_{\text{weight of each DnaA-ATP state}} \ , \tag{23}$$

where the exponential terms are the Boltzmann weights.

Thus, under the assumption that DnaA-ATP acts as an autorepressor, the total statistical weight of all the scenarios is given by

$$Z_{tot}(P,A) = \underbrace{Z(P,A)}_{\text{empty sites}} + \underbrace{Z(P-1,A)e^{-\varepsilon^S_{pd}/k_BT}}_{\text{RNAP on promoter}} + \underbrace{Z(P,A-1)e^{-\varepsilon^S_{ad}/k_BT}}_{\text{DnaA-ATP on specific site}}. \tag{24}$$

The probability that RNAP is bound at the *dnaA* promoter is

$$\mathbb{P}(\text{RNAP bound}) = \frac{Z(P-1,A)e^{-\varepsilon^S_{pd}/k_BT}}{Z_{tot}(P,A)} = \frac{1}{1+\frac{N_{NS}}{P}e^{\Delta\varepsilon_{pd}/k_BT}+\frac{A}{P}e^{(\Delta\varepsilon_{pd}-\Delta\varepsilon_{ad})/k_BT}}, \tag{25}$$

where it has been assumed that $N_{NS} \gg P$ and A.

The basal rate of transcription of the *dnaA* promoter is then defined as k_A and the copy-number of *dnaA* promoters in a given cell at one time (which can be computed from the Cooper-Helmstetter model) as $\Theta(t,\tau)$.

The same kind of argument can be used in order to justify the fact that one expects a constant initiation threshold for DnaA. Consider the fact that 20 DnaA molecules must bind to the origin for initiation to occur. It can be assumed that this state is binary, i.e. that either 20 molecules are bound, or 20 molecules are not bound. The probability of having 20 molecules bound is thus:

$$\mathbb{P}(\text{Origin Bound}) = \frac{Z(A-20)e^{-20\varepsilon_{ao}/k_BT}\omega}{Z(A-20)e^{-20\varepsilon_{ao}/k_BT}\omega + Z(A)},$$

where

$$Z(A) = \frac{N_{NS}!}{A!(N_{NS}-A)!}.$$

Dividing through, and under the assumptions above, gives that

$$\mathbb{P}(\text{Origin Bound}) = \frac{1}{1+\frac{e^{20\Delta\varepsilon_{ao}/k_BT}}{\omega(\kappa r)^{20}}}. \tag{26}$$

Thus, there is a value of r, determined by $\frac{e^{20\Delta\varepsilon_{ao}/k_BT}}{\omega\kappa^{20}}$ at which the probability rapidly approaches unity. This can be seen as the value of r where initiation takes place. Note that this estimate is rather robust to the addition of other possible states, such as states in which DnaA-ATP can be bound to other sites, assuming that the number of these extra specific sites is small in comparison to the total pool of DnaA-ATP molecules.

Section 2.1 Considerations on the increase in RNAP levels

A further factor to consider is the way in which RNAP levels vary during the cell cycle. As with other elements of the cell, the number of RNAP molecules must double during each cell cycle when the bacteria are in exponential growth. The assumption made here is that the RNAP levels grow exponentially during the cell cycle, to reach double their initial value at the end of the cell cycle. Combined with the assumption that the volume of the cells grows exponentially, this means that the concentration of RNAP stays constant through the cell cycle, in agreement with the results of Oeschger et al. [6]. Moreover, even if the levels of RNAP grow linearly, this is only a weak perturbation from the exponential growth. Consequently, the expectation is that this will not significantly affect the model dynamics.

Section 3 Parameter transformation enforcing a constant threshold

This section discusses the parameter transformation of equation (11), providing the key to probing how the parameters of the model must vary with growth rate. As discussed in the main text, equation (11) is transformed to fix the value of r at the moment of initiation ($t = X$) to be the same for every value of τ in the range $20 < \tau < 60$. This is enforced by a translation and scaling on r such that

$$r'(X,\tau) = \lambda(\tau) r(X,\tau) - a(\tau) \ , \tag{27}$$

where r' is the translated version of r.

The wish is to see what must happen to the coefficients in the equation for the fixed threshold condition to hold, when the structure of (11) is preserved in the transformation. The same transformation can be applied in numerical simulations, but its analytical form is more instructive with respect to classifying the possible parameter transformations.

Equation (11) can be rewritten in the desired form, with translated variables, as

$$\frac{\partial r'}{\partial t} = \frac{1}{\Lambda}\left(\frac{\Theta k'_A}{1 + c'_1 \frac{\Lambda}{P'} + c'_2 \frac{\Lambda}{P'} r'} - (k'_R + r' k_\Lambda)\mathcal{F}\right) \ . \tag{28}$$

Note that k_Λ cannot change, as this rate is externally imposed by the Cooper-Helmstetter model.

The substitution of r from (27) into equation (11) gives

$$\frac{\partial r'}{\partial t} = \lambda \frac{\partial r}{\partial t} = \frac{\lambda}{\Lambda}\left(\frac{\Theta k_A}{1 + c_1\frac{\Lambda}{P} + c_2\frac{\Lambda}{P}(\frac{r'+a}{\lambda})} - (k_R + \left(\frac{r'+a}{\lambda}\right)k_\Lambda)\mathcal{F}\right). \tag{29}$$

Comparison of the coefficients of r' and Λ in equation (29) with equation (28), yields the conditions

$$k'_A = \lambda k_A, \tag{30}$$

$$\lambda k_R + a k_\Lambda = k'_R, \tag{31}$$

$$\frac{c_2}{P\lambda} = \frac{c'_2}{P'}, \tag{32}$$

$$\frac{1}{P}\left(c_1 + c_2\frac{a}{\lambda}\right) = \frac{c'_1}{P'}, \tag{33}$$

and the condition that defines the transformation, namely

$$r'(X) = \lambda r(X) - a = constant. \tag{34}$$

At this point we note the assumption that the concentration of RNAP is constant throughout the cell cycle. Thus

$$P = P_0 e^{\alpha t} \tag{35}$$

and

$$P' = P'_0 e^{\alpha t} \tag{36}$$

Thus, since equations (32) and (33) have $e^{\alpha t}$ as a common factor, it is possible to divide through by it. From hereafter in this section, P and P' actually refer to P_0 and P'_0 but the subscripts are dropped for ease of notation.

Furthermore, equations (32) and (33) can combine to give

$$a = \frac{c'_1}{c'_2} - \lambda\frac{c_1}{c_2} \tag{37}$$

Examination of equation (4), reveals that

$$c_1 = \frac{b_0}{\kappa}$$
$$c_2 = \frac{b_0}{b_1} \tag{38}$$

where b_0 and b_1 are given by equation (4). Thus, writing $c_1' = \frac{b_0'}{\kappa}$ and $c_2' = \frac{b_0'}{b_1'}$ one sees that equation (37) can be rewritten

$$a = \frac{1}{\kappa}(b_1' - \lambda b_1) \tag{39}$$

where $b_1 = e^{\Delta\varepsilon_{ad}/k_B T}$.

Now, mathematically, two things can be chosen to be fixed to satisfy these equations

1. The RIDA rate can be fixed: $k_R = k_R'$. It then follows from (31) that

$$a = \frac{k_R}{k_\Lambda}(1 - \lambda), \tag{40}$$

which, when combined with equation (39) gives

$$b_1' = \frac{\kappa k_R}{k_\Lambda}(1 - \lambda) + \lambda b_1. \tag{41}$$

Equation (40), when taken with (34), gives the value that λ must take, namely

$$\lambda = \frac{r'(X) + \frac{k_R}{k_\Lambda}}{r(X) + \frac{k_R}{k_\Lambda}}. \tag{42}$$

Either c_1 (i.e. b_0) or P can then be fixed.

(a) Since it is possible to choose c_1', it is logical to set $c_1' = c_1$. This gives that, $c_2' = \frac{b_0}{b_1'}$ and so

$$P' = \frac{b_1 P \lambda}{\left(\kappa \frac{k_R}{k_\Lambda}(1 - \lambda) + \lambda b_1\right)}. \tag{43}$$

Note that any trend for b_0 could have been chosen. The decision to set $b_0' = b_0$ here, was due to it being the simplest logical choice. This equation, taken with equations (30), (41) and (42) determines the transformation.

(b) It is possible to fix P' to a known trend e.g. the one given in ref. [7]. This will then have implications for how c_1 (and hence b_0) must vary. Given that P' is now a known quantity, this gives that

$$b_0' = \frac{b_0 P' \left(\kappa \frac{k_R}{k_\Lambda}(1 - \lambda) + \lambda b_1\right)}{b_1 P \lambda}. \tag{44}$$

This equation, taken with equations (30), (41) and (42) determines the transformation.

2. It is possible to set $c_1 = c_1'$ and $c_2 = c_2'$. From this it follows that

$$a = \frac{c_1}{c_2}(1 - \lambda) \tag{45}$$

and

$$P' = \lambda P. \tag{46}$$

Furthermore,

$$k_R' = \frac{k_R - \frac{c_1}{c_2}(1 - \lambda)k_\lambda}{\lambda}. \tag{47}$$

Thus, equations (45), (46) and (47), along with

$$\lambda = \frac{r'(X) + \frac{c_1}{c_2}}{r(X) + \frac{c_1}{c_2}} \tag{48}$$

and (30), determine the transformation, when c_1 and c_2 are fixed.

Section 4 Average over cell population

This section discusses how to compute average levels of RNA polymerase and average gene expression, used for example in Figure 4. These average levels are quantities that can be in principle measured directly.

Consider, for example, the expression level of the *dnaA* promoter (i.e. Q) (presented in Figure 4B). Denote the average colony expression level as:

$$\text{Average colony expression level } = \langle Q(\alpha) \rangle \tag{49}$$

where steady exponential growth is assumed and α is the growth rate. In order to evaluate this average, the probability of finding a cell at a time $t \in (0, \tau)$ of the cell cycle is required.

Given the exponentially growing colony with growth rate α, let $N(t)$ denote the number of bacteria in the colony at time t. If the interval $(0, \tau)$ is split into $\tau/\delta t$ equal size segments each of infinitesimal size δt, then the number of bacteria born in the infinitesimal time inverval $(t, t + \delta t)$ is

$$\delta N(t) = \frac{\mathrm{d}N}{\mathrm{d}t}\delta t \ , \tag{50}$$

i.e.

$$\delta N(t) = \alpha N \, \mathrm{d}t = \alpha N_0 e^{\alpha t} \delta t. \tag{51}$$

Within a population of bacteria at time t', the probability of finding a bacterium born in the time interval $(t_b, t_b + \delta t)$ (with $t_b < t'$) is given by

$$\frac{\delta N(t_b)}{N(t')} = \frac{\alpha N_0 e^{\alpha t_b}}{N(t')} \delta t = \alpha e^{\alpha (t_b - t')} \delta t. \tag{52}$$

This is equivalent to finding a bacterium with an age in the range $(t, t + \delta t)$ where $t = t' - t_b$, and so it can be written that the probability of finding a bacterium with an age in the range $(t, t + \delta t)$ is

$$\mathcal{P}(t, \alpha) = \alpha e^{-\alpha t} \delta t. \tag{53}$$

Moreover, since bacteria divide after a time τ, the probability of finding a bacterium at a stage in the range $(t, t + \delta t)$ of the cell cycle is given by

$$\begin{aligned}
\mathbb{P}(t, \alpha) &= \sum_{n=0}^{\infty} \mathcal{P}(n\tau + t) \\
&= \alpha e^{-\alpha t} \sum_{0}^{\infty} e^{-\alpha n \tau} \delta t \\
&= \alpha e^{-\alpha t} \left(\frac{1}{1 - e^{-\alpha \tau}} \right) \delta t \\
&= \alpha e^{-\alpha t} \left(\frac{1}{1 - \frac{1}{2}} \right) \delta t \\
&= 2\alpha e^{-\alpha t} \delta t \\
&= \phi(t, \alpha) \delta t
\end{aligned} \tag{54}$$

Note that the individual bacteria are assumed to grow exponentially with growth rate α in the same way as the colony itself. Thus,

$$\begin{aligned}
\langle Q(\alpha) \rangle &= \sum_{m=0}^{\tau/\delta t} Q(m\delta t, \alpha) \mathbb{P}(m\delta t, \alpha) \\
&= \sum_{m=0}^{\tau/\delta t} Q(m\delta t, \alpha) \phi(m\delta t, \alpha) \delta t \\
&= \int_0^{\tau} Q(t, \alpha) \phi(t, \alpha) \, \mathrm{d}t \qquad \text{in the limit as } \delta t \to 0
\end{aligned} \tag{55}$$

This argument gives the average value of any observable quantity. For example to get the average of P, as in Figure 4A, one would take

$$\langle P(\alpha)\rangle = \int_0^{\tau} P(t,\alpha)\phi(t,\alpha)\,\mathrm{d}t. \tag{56}$$

Section 5 Considerations on other forms of the promoter

This section deals with thermodynamic models for promoters that incorporate some further experimental findings on the behaviour of the $dnaA$ gene [8]. Data from footprint experiments has provided an insight into the cooperativity of the DnaA boxes on the $dnaA$ gene, which has been proposed to be important for the correct timing of DNA replication in *Escherichia coli*, as well as potential autoactivation of the gene [9]. The models considered are (i) a promoter with no autorepression, (ii) a model with cooperativity of DnaA binding to the repression sites at the *dnaA* promoter, (iii) a promoter with both cooperativity and autoactivation. The reasons for not choosing these model variants as the main model formulation are then discussed.

Section 5.1 Promoter with no autorepression

This section considers a simpler model for the promoter than the one used in the main text, namely one that has no autorepression. The form for this type of promoter was derived in Section 2 and its implications are discussed more fully here. When the results from Section 2 are used, the main equation takes the form

$$\frac{\partial r}{\partial t} = \frac{1}{\Lambda}\left(\frac{\Theta k_A}{1 + c_1\frac{\Lambda}{P}} - (k_R + r k_\Lambda)\mathcal{F}\right). \tag{57}$$

This is integrated, with fixed parameters k_A, c_1, k_R and k_Λ assumed, to give $r(t,\tau)$, for a given τ. The parameter c_1 is then varied (for a fixed value of τ) to probe the effect that autorepression has (see Figure A2). What can be see in Figure A2 is that autorepression causes the range of values for $r(t,\tau)$ (the oscillation size), to be much narrower than for the non-autorepressed case. This might be an important role that autorepression plays in fine tuning the timing of replication initiation, since it ensures that, even before any other parameters are considered to be variable, the differences in the ratio DnaA-ATP:DNA length is reduced across different τ. For this reason, autorepression was chosen to be included in the main model formulation.

Section 5.2 Promoter with cooperativity of DnaA binding

This section discusses a model for a promoter in which there are two binding sites for DnaA. First, it deals with a form for a simple case of cooperativity, and then it introduces a more complex form including differential action of

DnaA-ATP and DnaA-ADP.

Section 5.2.1 Simple cooperativity

In the model considered here, there are two binding sites for DnaA, both of which have a repression effect when bound, and which bind highly cooperatively. The sites are considered to have a low affinity for DnaA so only a probability to the situation in which both sites are bound is allocated . As in Section 2, the number of DnaA-ATP molecules is denoted as A, the number of non-specific binding sites as N_{NS} and the number of RNA polymerase molecules as P. Furthermore, the binding energy between a molecule x and a (non-)specific site on the DNA is defined as $\varepsilon_{xd}^{(N)S}$ and the difference is denoted as

$$\Delta\varepsilon_{xd} = \varepsilon_{xd}^{S} - \varepsilon_{xd}^{NS} . \tag{58}$$

In addition, the cooperativity of binding between two DnaA-ATP molecules is written as ω.
Thus, the sum of all the statistical weights is

$$Z_{tot} = \underbrace{Z(P,A)}_{\text{Empty sites}} + \underbrace{Z(P-1,A)e^{-\varepsilon_{pd}^{S}}}_{\text{RNAP bound}} + \underbrace{Z(P,A-2)e^{-2\varepsilon_{ad}^{S}}\omega}_{\text{DnaA-ATP bound cooperatively}} . \tag{59}$$

This form allocates zero probability that DnaA-ATP can bind by itself. This leads directly to the probability of RNAP being bound to the promoter, namely

$$\mathbb{P}(\text{RNAP bound}) = \frac{Z(P-1,A)e^{-\varepsilon_{pd}^{S}}}{Z_{tot}} , \tag{60}$$

i.e.

$$\mathbb{P}(\text{RNAP bound}) = \frac{1}{1 + e^{\Delta\varepsilon_{pd}} \frac{N_{NS}}{PF_{reg}}} , \tag{61}$$

where

$$F_{reg}^{-1} = 1 + e^{-2\Delta\varepsilon_{ad}}\omega \left(\frac{A}{N_{NS}}\right)^2 . \tag{62}$$

Figure A6C shows that the presence of cooperativity alone cannot produce a constant threshold. Thus, it is necessary to try and impose a constant threshold on the model using a transformation such as that described in Section 3. Now, if it is written that $r\kappa = \frac{A}{N_{NS}}$ (see main text for definition of κ), an attempt to perform the same transformation as in Section 3, i.e.

$$r' = \lambda r - a , \tag{63}$$

shows that the $(r\kappa)^2$ term produces a linear term in r' that is not present in the original form of F_{reg} and so the transformation will fail as it will not generally be possible to retain the original form of the model.

Section 5.2.2 A more sophisticated model for cooperativity

We consider here the more realistic situation where the two binding sites differ. One is a high-affinity site, which does not contribute towards autorepression, and the other is a low-affinity site to which DnaA binds cooperatively and such that, when bound, DnaA has a repression effect on the *dnaA* gene [10]. The number of ways of distributing P RNA polymerase molecules and A DnaA-ATP molecules in N_{NS} binding sites is thus

$$Z(P,A) = \frac{N_{NS}!}{P!A!(N_{NS}-P-A)} \times e^{-P\varepsilon_{pd}^{NS}} e^{-A\varepsilon_{ad}^{NNS}} . \quad (64)$$

There are five different possible states of the system, described in the sum of the statistical weights:

$$\begin{aligned} Z_{tot} = &\underbrace{Z(P,A)}_{\text{Empty sites}} \\ &+ \underbrace{Z(P-1,A)e^{-\varepsilon_{pd}^S}}_{\text{RNAP bound}} \\ &+ \underbrace{Z(P-1,A-1)e^{-(\varepsilon_{pd}^S+\varepsilon_{ad}^H)}}_{\text{RNAP bound and high affinity site bound}} \\ &+ \underbrace{Z(P,A-1)e^{-\varepsilon_{ad}^H}}_{\text{High affinity site bound and RNAP not bound}} \\ &+ \underbrace{Z(P,A-2)e^{-(\varepsilon_{ad}^H+\varepsilon_{ad}^L)}\omega}_{\text{DnaA-ATP bound cooperatively}} . \end{aligned} \quad (65)$$

This model allocates zero probability that DnaA-ATP can bind to the low-affinity site alone. For it to bind to this site it must do so cooperatively once the high affinity site is bound.

This leads to the probability of RNAP being bound to the promoter, namely:

$$\mathbb{P}(\text{RNAP bound}) = \frac{Z(P-1,A)e^{-\varepsilon_{pd}^S} + Z(P-1,A-1)e^{-(\varepsilon_{pd}^S+\varepsilon_{ad}^H)}}{Z_{tot}} , \quad (66)$$

i.e.

$$\mathbb{P}(\text{RNAP bound}) = \frac{1}{1+e^{\Delta\varepsilon_{pd}}\frac{N_{NS}}{PF_{reg}}} \quad (67)$$

where

$$F_{reg}^{-1} = \frac{1 + \frac{A}{N_{NS}} e^{-\Delta\varepsilon_{ad}^{H}} + \omega \left(\frac{A}{N_{NS}}\right)^2 e^{-(\Delta\varepsilon_{ad}^{H} + \Delta\varepsilon_{ad}^{L})}}{1 + \frac{A}{N_{NS}} e^{-\Delta\varepsilon_{ad}^{H}}}. \tag{68}$$

Now, writing $r\kappa = \frac{A}{N_{NS}}$ (see main text for definition of κ), the same transformation as for the main equation can be performed, following the steps in Section 3, i.e. writing

$$r' = \lambda r - a \tag{69}$$

and collecting and comparing terms in r'.

Section 5.3 Promoter with both cooperativity and autoactivation

This section considers an even more realistic form for the promoter which incorporates both autoactivation and cooperativity of DnaA binding of both forms of bound DnaA. The main conclusion is that this representation introduces too many new parameters in the model, which cannot be estimated from experiments, and therefore adds uncontrolled uncertainty. Hence, priority was given to the simpler and more controlled model presented in the main text.

First, consider the $dnaA$ gene itself, which has in fact two promoters, $dnaAp1$ and $dnaAp2$. Since the expression of the $dnaA$ gene is controlled mostly by the $dnaAp2$ promoter, only this promoter is considered. There are 5 DnaA boxes on the $dnaA$ gene, two high affinity boxes (1 and 2) which bind both DnaA-ATP and DnaA-ADP and three low affinity boxes (a, b and c) which bind only DnaA-ATP. Since box a appears to only affect the $dnaAp1$ promoter, it is ignored for this derivation. DnaA boxes b and c appear to cause autorepression when bound by DnaA-ATP. Boxes 1 and 2 appear to cause autoactivation when bound by either DnaA-ATP or DnaA-ADP in situations when DnaA boxes b and c are unbound. Moreover, it is assumed that, due to cooperativity, there must be pairwise binding between boxes 1 and 2 and boxes b and c i.e. if box 1 is bound by DnaA-ATP then box 2 must also be bound by DnaA-ATP [9].

In a similar manner to the previous section, the number of DnaA-ATP molecules is denoted as A_-, the number of DnaA-ADP molecules as A_+, the number of non-specific binding sites as N_{NS} and the number of RNA polymerase molecules as P. Furthermore, the binding energy between a molecule x and a (non-)specific site, i, on the DNA is defined as $\varepsilon_{xd}^{(N)S_i}$ and the difference as

$$\Delta\varepsilon_{xd} = \varepsilon_{xd}^{S_i} - \varepsilon_{xd}^{NS} . \tag{70}$$

In addition to this, the binding energy (cooperativity) between two molecules a and b is written as ε_{ab}. So, the statistical weight of distributing P RNA polymerase molecules, A_- DnaA-ATP molecules and A_+ DnaA-ADP molecules among N_{NS} non-specific binding sites is

$$Z(P, A_-, A_+) = \frac{N_{NS}!}{P!A_-!A_+!(N_{NS} - P - A_- - A_+)} \times e^{-P\varepsilon_{Pd}^{NS}} e^{-A_-\varepsilon_{A_-d}^{NS}} e^{-A_+\varepsilon_{A_+d}^{NS}} . \tag{71}$$

The sum of all the statistical weights is

$$\begin{aligned} Z_{tot} = {} & Z(P, A_-, A_+) + Z(P-1, A_-, A_+)e^{-\varepsilon_{Pd}^{S}} \\ & + Z(P, A_- - 2, A_+)k_{b,c} + Z(P-1, A_- - 2, A_+)e^{-\varepsilon_{Pd}^{S}} k_{1,2}^{-}\omega_{0,1}^{-} \\ & + Z(P, A_- - 2, A_+)k_{1,2}^{-} + Z(P, A_-, A_+ - 2)k_{1,2}^{+} \\ & + Z(P, A_- - 4, A_+)k_{b,c}k_{1,2}^{-}\omega_{b,1}^{-} + Z(P-1, A_-, A_+ - 2)e^{-\varepsilon_{Pd}^{S}} k_{1,2}^{+}\omega_{0,1}^{+} \\ & + Z(P, A_- - 2, A_+ - 2)k_{b,c}k_{1,2}^{+}\omega_{b,1}^{+}, \end{aligned} \tag{72}$$

where

$$\begin{aligned} k_{b,c} &= e^{-2\varepsilon_{A_-d}^{S_{b,c}}} \\ k_{1,2}^{-} &= e^{-2\varepsilon_{A_-d}^{S_{1,2}}} \\ k_{1,2}^{+} &= e^{-2\varepsilon_{A_+d}^{S_{1,2}}} \\ \omega_{0,1}^{-} &= e^{-\varepsilon_{A_-P}} \\ \omega_{0,1}^{+} &= e^{-\varepsilon_{A_+P}} \\ \omega_{b,1}^{+} &= e^{-\varepsilon_{A_-A_+}^{b,1}} \\ \omega_{b,1}^{-} &= e^{-\varepsilon_{A_-A_-}^{b,1}} . \end{aligned} \tag{73}$$

Thus, the probability of RNA polymerase being bound to the *dnaA* promoter is

$$\begin{aligned} \mathbb{P}(\text{RNAP bound}) = \frac{1}{Z_{tot}} \Big(& Z(P-1, A_-, A_+)e^{-\varepsilon_{Pd}^{S}} + Z(P-1, A_- - 2, A_+)e^{-\varepsilon_{Pd}^{S}} k_{1,2}^{-}\omega_{0,1}^{-} \\ & + Z(P-1, A_-, A_+ - 2)e^{-\varepsilon_{Pd}^{S}} k_{1,2}^{+}\omega_{0,1}^{+} \Big) , \end{aligned} \tag{74}$$

i.e.

$$\mathbb{P}(\text{RNAP bound}) = \frac{1}{1 + e^{\Delta\varepsilon_{Pd}} \frac{N_{NS}}{PF_{reg}}}, \tag{75}$$

where

$$F_{reg}^{-1} = \frac{1 + \Delta k_{b,c}\left(\frac{A_-}{N_{NS}}\right)^2 + \Delta k_{1,2}^-\left(\frac{A_-}{N_{NS}}\right)^2 + \Delta k_{1,2}^+\left(\frac{A_+}{N_{NS}}\right)^2 + \Delta k_{b,c}\Delta k_{1,2}^-\omega_{b,1}^-\left(\frac{A_-}{N_{NS}}\right)^4 + \Delta k_{b,c}\Delta k_{1,2}^+\frac{A_-^2 A_+^2}{N_{NS}^4}}{1 + \Delta k_{1,2}^-\omega_{0,1}^-\left(\frac{A_-}{N_{NS}}\right)^2 + \Delta k_{1,2}^+\omega_{0,1}^+\left(\frac{A_+}{N_{NS}}\right)^2}, \tag{76}$$

where it has been assumed that $N_{NS} \gg P$, A_- and A_+. In this equation, $\Delta k_{i,j}^{(+/-)} = k_{i,j}^{(+/-)} \times e^{2(\varepsilon_{A_{+/-}}^{NS} - d)}$.
Now, if the substitution $r\kappa = \frac{A_-}{N_{NS}}$ is used (see main text for definition of κ), then when a transformation such as that in Section 3, namely

$$r' = \lambda r - a, \tag{77}$$

is attempted, it can be seen that the $(r\kappa)^2$ and $(r\kappa)^4$ terms will create terms that are linear, and cubic, in r'. Thus, it is not possible in general to make a scaling and translation on r in this way and keep the F_{reg} in the same form. This information shows that this model of the promoter, with fixed parameters, cannot explain a constant threshold for the ratio r at initiation, and suggests that analyzing this promoter poses a different problem altogether as it cannot be done by applying the techniques used in this work for the more simple promoter. Moreover, equation (73) introduces many new parameters in the model, which generally cannot be directly estimated from experiments, and therefore adds an element of uncertainty into the model. For this reason it is believed that, while the promoter model is satisfactory with respect to existing footprinting data [9] , in absence of more precise knowledge, a simple controlled model such as the one presented in the main text is to be preferred.

Section 6 Considerations on other model ingredients

Section 6.1 DnaA-ATP recycling regions

As explained in the main text, the DnaA recycling sequences, known to convert the ADP-bound form of DnaA into its ATP-bound form were considered in a model variant. Here we discuss the mathematics of including a term in the equation to represent this reactivation. We show how it counters RIDA, with a rate that is proportional to the genome amount rather than genome replication rate, and analyze its effects under variations of the growth rate.
The number of the DnaA recycling sites is assumed to be proportional to the total length of the genome in the cell. Furthermore, regeneration is assumed to be replication fork independent, and it is assumed that the rate limiting

parameter is the recycling rate per recycling site, rather than the amount of DNA-ADP in the cell. Thus the new, effective, RIDA term becomes:

$$\tilde{k_R} = k_R - \rho\frac{\Lambda}{\mathcal{F}}, \tag{78}$$

where ρ is the recycling rate per length of chromosome (i.e. ρ is (the recycling rate per site)$\times$(the number of recycling sites per genome equivalent). Performing the same transformation as in equation (27) gives

$$k'_R = \lambda k_R + ak_\Lambda \tag{79}$$

and

$$\rho' = \lambda\rho \tag{80}$$

i.e. unless $\lambda = 1$, the recycling rate per site must also vary with growth rate. This indicates that the effect of DARS recycling is not able by itself to impose a constant threshold with varying growth rate.

Note that one now has

$$\tilde{k}'_R = \lambda\tilde{k}_R + ak_\Lambda\ , \tag{81}$$

which is equivalent to equation (31) with $\tilde{k}_R$ in place of k_R. Thus this new, effective RIDA rate behaves similarly as the previous RIDA rate does in the main equation, scaling and translating as a function of cell doubling time.

Section 6.2 Specific binding sites for DnaA

This section considers a model variant including specific sites along the chromosome, at which DnaA-ATP can bind, providing a titrating effect reducing the reservoir of free DnaA-ATP that can be bound non-specifically on the chromosome. We show that the inclusion of a term of this form is equivalent to a reduction of the RIDA rate.

Let β denote the number of specific binding sites per genome equivalent. We further assume that once these specific binding sites are created, they are (nearly) always bound by a DnaA-ATP molecule. Thus

$$\begin{aligned}\frac{\partial A_-}{\partial t} &= Q - k_R\mathcal{F} - \beta\frac{\partial\Lambda}{\partial t}\\ &= Q - (k_R + \beta k_\Lambda)\mathcal{F},\end{aligned} \tag{82}$$

where A_- now represents the 'free' DnaA-ATP. Thus, equation (82) is the same as the main equation for $\frac{\partial A_-}{\partial t}$, but with an effective increase of the RIDA rate due to titration, where the new, effective, RIDA rate is given by

$$\tilde{k}_R = k_R + \beta k_\Lambda. \tag{83}$$

$\beta \approx 300$ and $k_\Lambda = \frac{1}{40}\text{min}^{-1}$, hence $\beta k_\Lambda \approx 7.5\text{min}^{-1}$. As shown in Additional Figure A8, the model is robust to changes in the RIDA rate of this order of magnitude, and thus adding the role of specific binding sites does not affect the qualitative behavior of the model.

Section 7 Experimental Methods

Escherichia coli K-12 strains BW25113 carrying the pKK-*gfp* plasmid, where the reporter gene *gfp* (green fluorescent protein) was expressed under control of the *dnaA* promoter region (PdnaA), were grown at 37°C in four different M9 minimal media to support different growth rates. Fluorescence and optical density were measured as a function of time with an automated temperature-controlled plate reader Wallac Victor3. Gene expression was calculated by taking the time derivative of the fluorescence divided by the optical density, which provides a measure of the promoter activity as previously described [11].

To obtain data for the promoter activity as a function of growth rate during exponential phase, an exponential window was automatically detected by taking as maximum value the inflection point of the OD curve and as minimal value the first OD value that is two times higher than the OD background value. In this window the data was then fitted to an exponential function and this fit was used in order to extract the values of generation time. Expression data for different growth rates were corrected for plasmid abundance.

Additional File 1 References

1. Donachie W: **Relationships between cell size and time of initiation of DNA replication**. *Nature* 1968, **219**:1077–1079.
2. Schaechter M, Maaloe O, Kjeldgaard NO: **Dependency on medium and temperature of cell size and chemical composition during balanced growth of *Salmonella typhimurium***. *Journal of General Microbiology* 1958, **19**(3):592–606.
3. Shea MA, Ackers GK: **The OR control system of bacteriophage lambda, a physical-chemical model for gene regulation**. *Journal of Molecular Biology* 1985, **181**:211–230.
4. Ackers GK, Johnson AD, Shea MA: **Quantative model for gene regulation by λ phage repressor**. *PNAS* 1982, **79**:1129–1133.
5. Bintu L, Buchler N, Garcia H, Gerland U, Hwa T, Kondev J, Phillips R: **Transcriptional regulation by the numbers: models**. *Current Opinion in Genetics and Development* 2005, **15**:116–124.
6. Oeschger MP, Berlyn MKB, Mar N: **Regulation of RNA polymerase synthesis in *Escherichia coli*: A mutant unable to synthesize the enzyme at 43 degrees**. *Sciences-New York* 1974, **72**(3):911–915.
7. Klumpp S, Hwa T: **Growth-rate-dependent partitioning of RNA polymerases in bacteria**. *PNAS* 2008, **105**:20245–20250.
8. Donachie W, Blakely G: **Coupling the initiation of chromosome replication to cell size in *Escherichia coli***. *Current Opinion in Microbiology* 2003, **6**:146–150.
9. Speck C, Weigel C, Messer W: **ATP- and ADP-DnaA protein, a molecular switch in gene regulation**. *The EMBO Journal* 1999, **18**:6169–6176.
10. Speck C, Messer W: **Mechanism of origin unwinding: sequential binding of DnaA to double- and single-stranded DNA**. *The EMBO Journal* 2001, **20**:1469–1476.
11. Zaslaver A, Kaplan S, Bren A, Jinich A, Mayo A, Dekel E, Alon U, Itzkovitz S: **Invariant distribution of promoter activities in Escherichia coli.** *PLoS Comput Biol* 2009, **5**(10):e1000545.

Section 8 Additional Figures

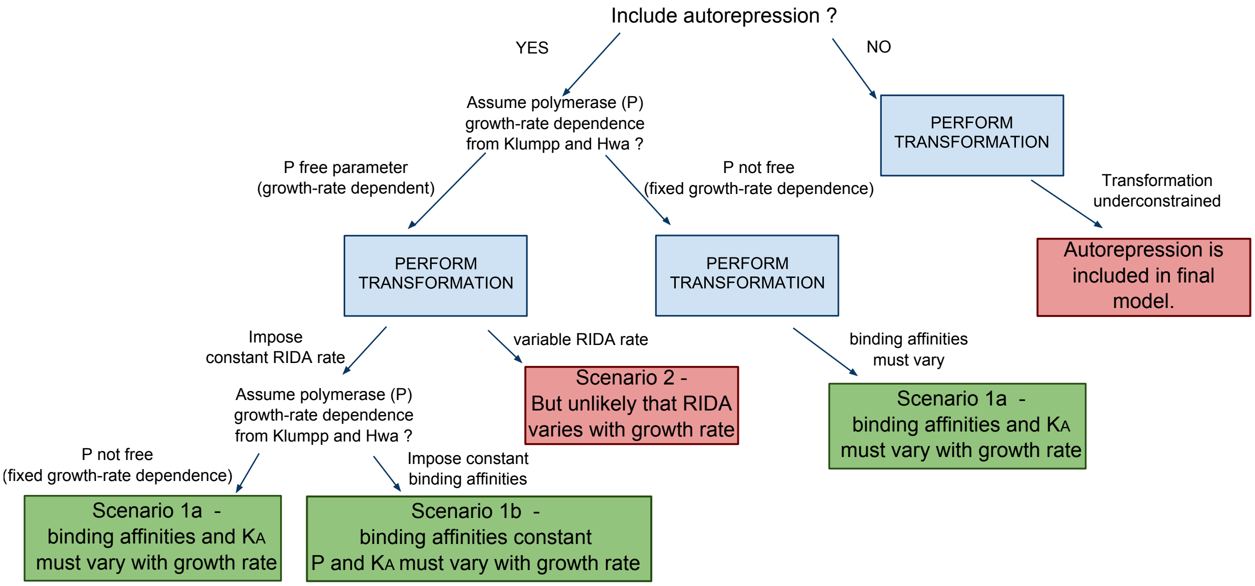

Figure A1: **Flow-chart of the procedure adopted for defining the scenarios of parameter variation with growth rate.**

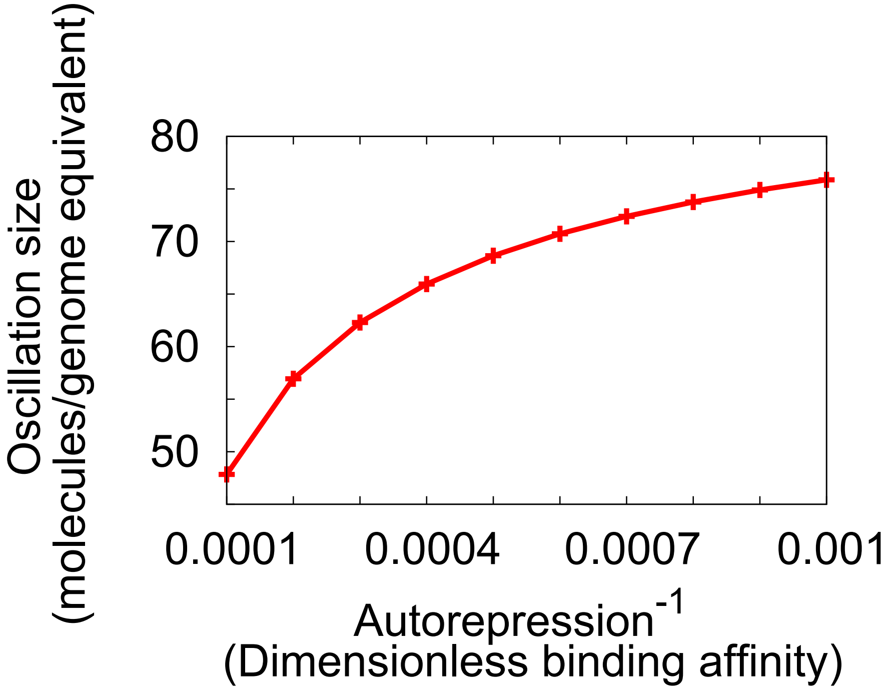

Figure A2: **Autorepression reduces the amplitude of the oscillations of the cell cycle.** If one varies the dimensionless binding affinity of DnaA-ATP to its self-repression sites, one can effectively vary the amount of autorepression. As the amount of autorepression is increased, the amplitude of the oscillations of the cell cycle decrease. The amplitude is defined as the difference between the maximum and minumum values of r in a given cell cycle. This cell cycle length in this plot is $\tau = 35$ mins.

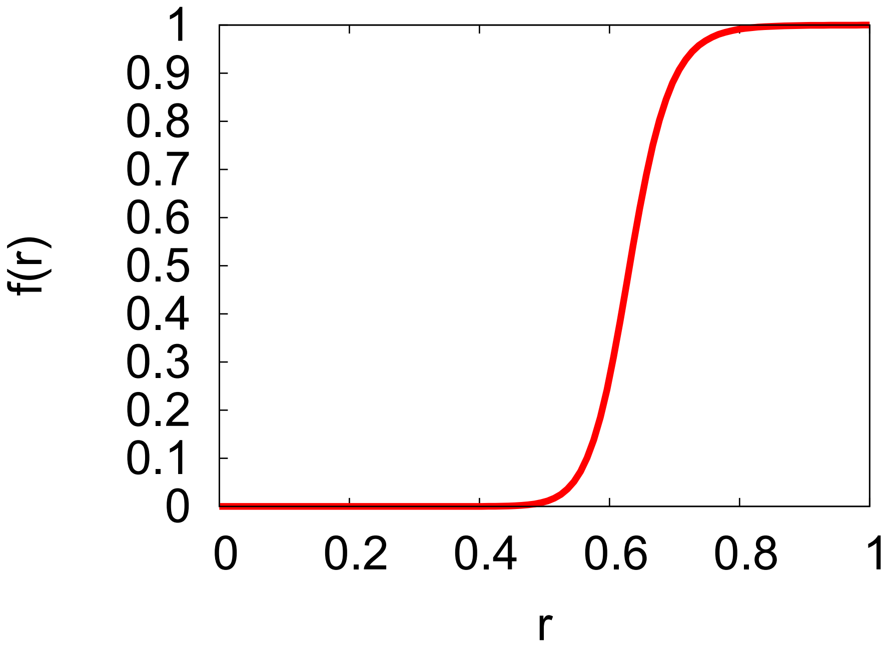

Figure A3: **There is a critical value at which the probability of DnaA-ATP molecules binding to the origin quickly approaches unity.** In this plot, $f(r) = \frac{1}{\omega e^{20\Delta\varepsilon/k_B T} r^{-20} + 1}$, which is the probability of having 20 DnaA-ATP molecules bound to the origin, where we have taken $\omega e^{20\Delta\varepsilon/k_B T} = 0.0001$ in this case.

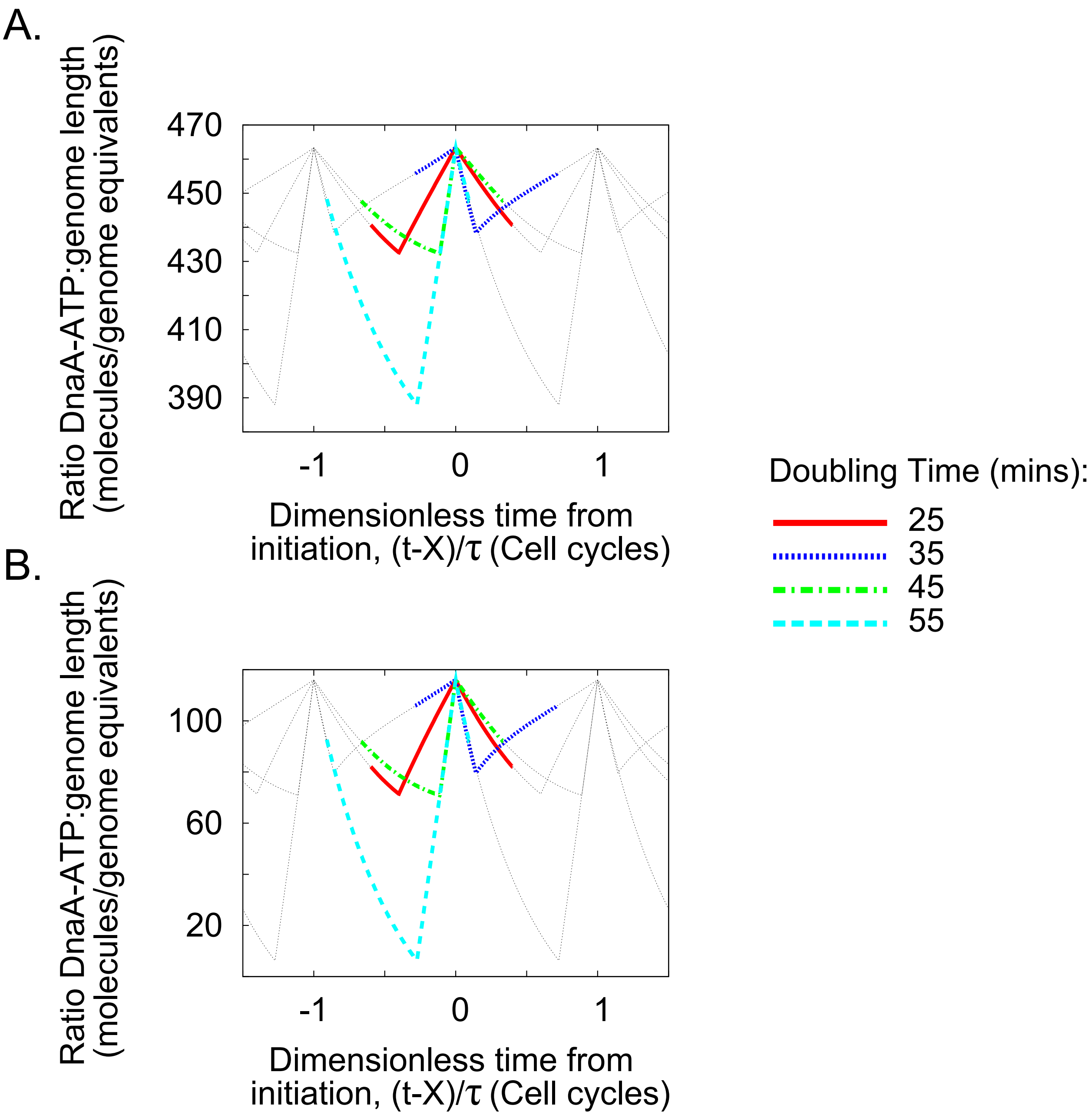

Figure A4: **A constant threshold can still be achieved when the RIDA rate k_R is varied significantly.** A: The oscillations of the cell cycle when $k_R = 2$ molecules/minute. B: The oscillations of the cell cycle when $k_R = 17$ molecules/minute. Despite RIDA rates being nearly 10 fold different, a constant threshold can still be achieved for each.

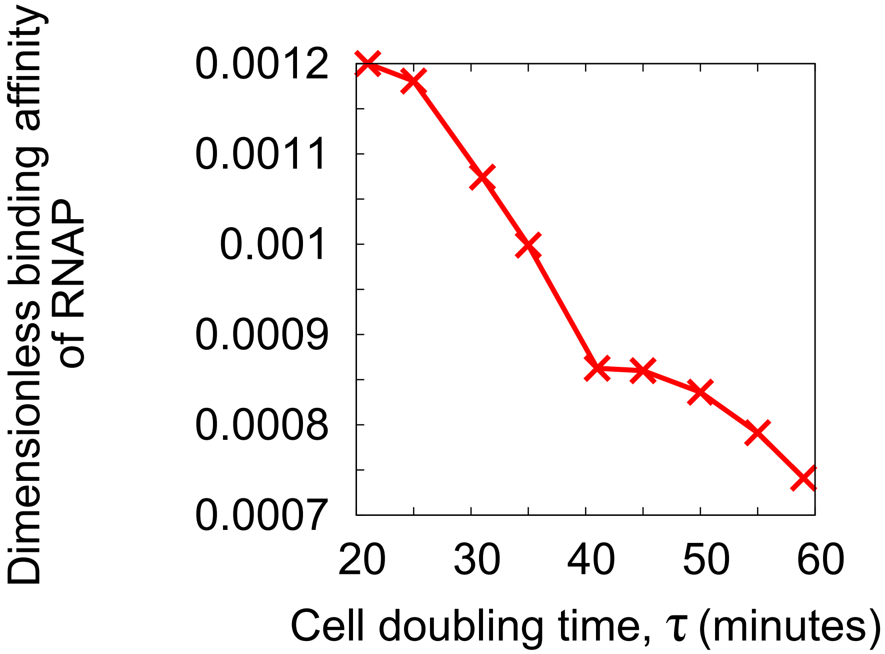

Figure A5: **In scenario 1a, the binding affinity of RNAP to the DnaA promoter ($e^{\Delta\varepsilon_{pd}/k_BT}$) varies with cell doubling time (τ).** In particular it decreases with cell doubling time, in a similar manner to the binding affinity of DnaA-ATP. This could be caused by supercoiling.

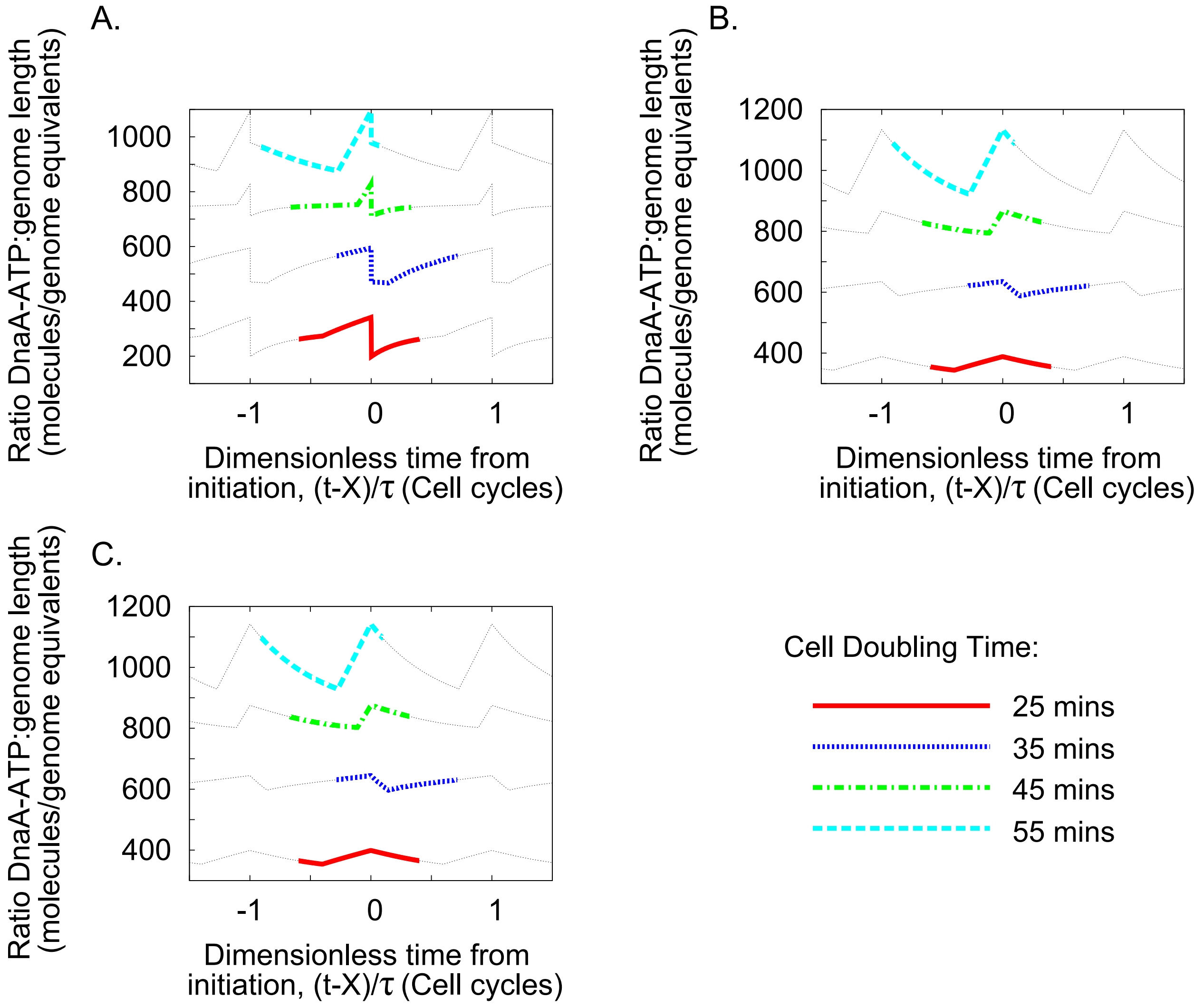

Figure A6: **Model variants were considered which all fail to explain a constant initiation threshold in r.** The models include: A: The *datA* locus, binding up to 300 DnaA-ATP molecules soon after initiation. B: A delay in the synthesis of DnaA-ATP, to reflect the time interval between the mRNA being transcribed and the DnaA protein being translated. C: A more complex form for the *dnaA* promoter, containing two binding sites for DnaA which binds cooperatively. None of these variants succeeds in achieving a constant initiation threshold in r, leading us to pursue a model in which some of the parameters of the model are able to vary with growth rate.

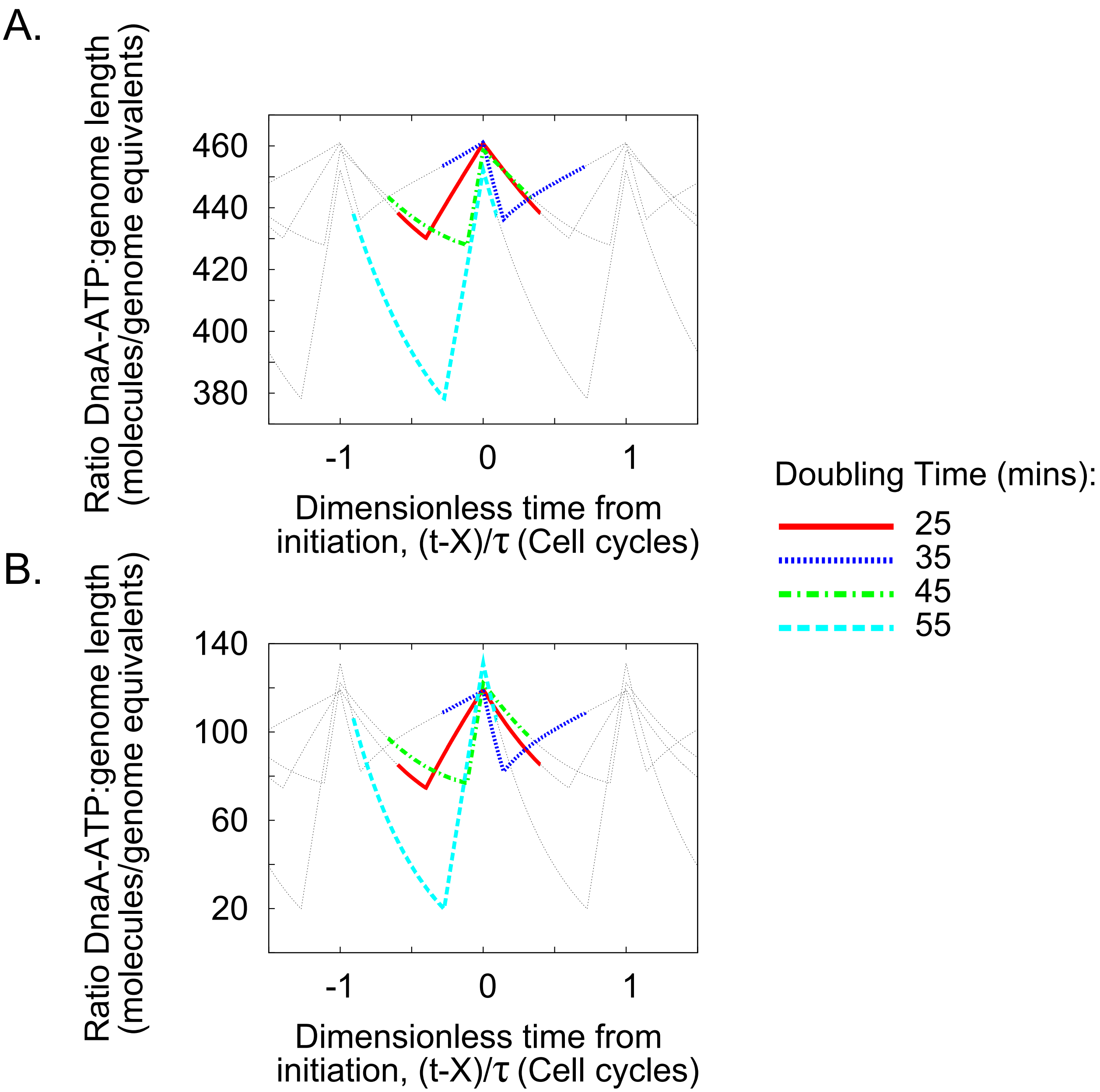

Figure A7: **A mutant in which the RIDA rate is varied.** Attempting to reproduce the effect of over or under-expressing the Hda protein or sequestering DnaA from specific titration sites uniformly distributed along the genome, we modified the RIDA rate leaving the other parameters fixed to their values when $k_R = 10$ molecules/minute. A: The RIDA rate is reduced to $k_R = 2$ molecules/minute, resulting in a lower value of r at $t = X$ at slower growth rates, and thus later initiation time. B: The RIDA rate is increased to $k_R = 17$ molecules/minute, resulting in a higher value of r at $t = X$ at slower growth rates, and thus earlier initiation time.

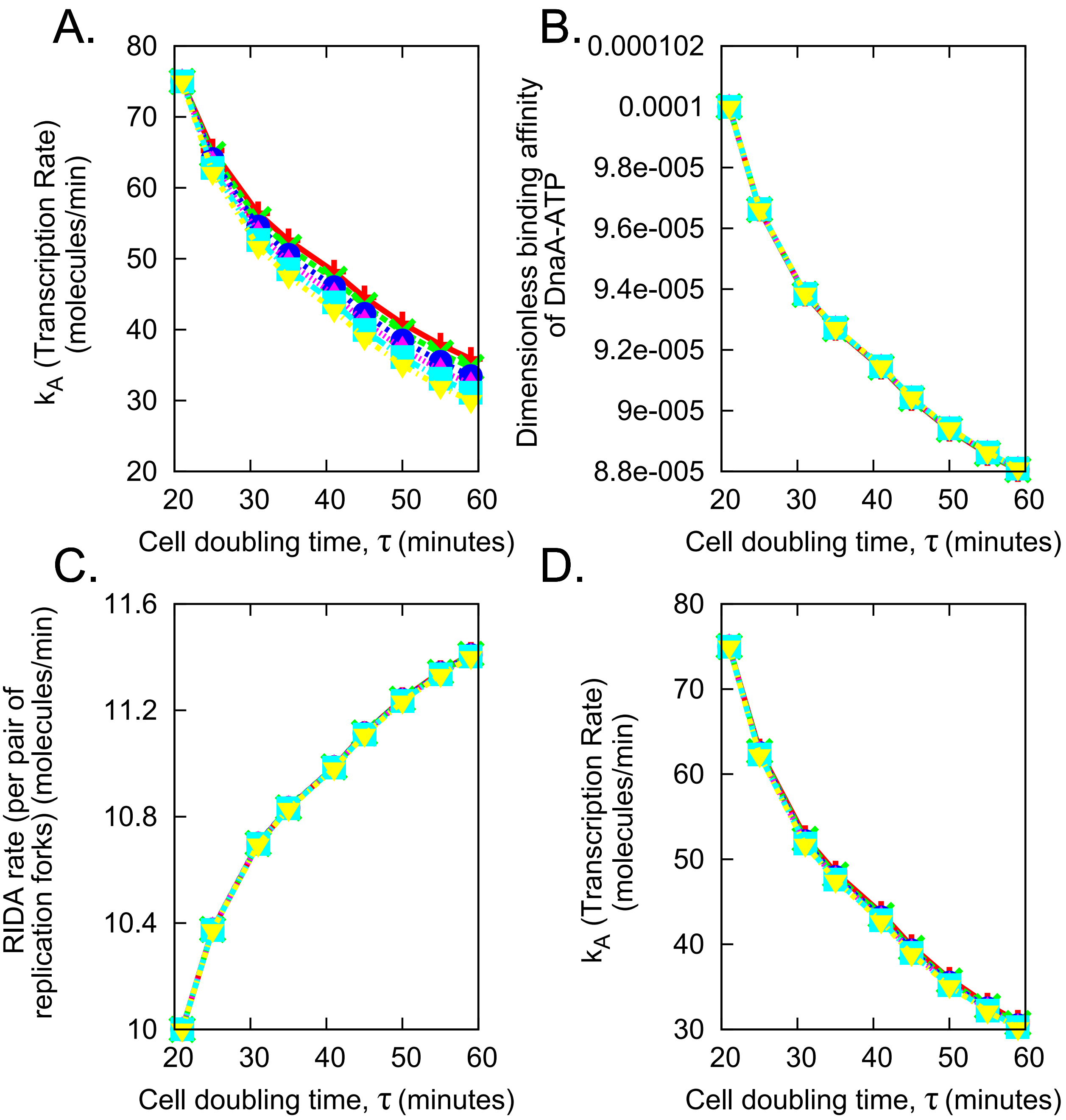

Figure A8: **The model is robust to changes in the input parameters.** In order to show that the qualitative trends, observed from transforming the main equation, were independent of the choice of parameters, the values of the input parameters were varied and the trends replotted. A: The trend of k_A as the value of the RIDA rate, k_R, is varied, with $k_R \in \{5, 7, 9, 11, 13, 15\}$, denoted by the symbols, +, ×, ●, ▲, ■, ▼ respectively. B: The trend of the dimensionless binding affinity of DnaA-ATP to its self-repression sites as k_A is varied, with $k_A \in \{50, 60, 70, 80, 90, 100\}$, denoted by the symbols, +, ×, ●, ▲, ■, ▼ respectively. C: The trend of the RIDA rate, k_R as the dimensionless binding affinity of RNAP to the DnaA promoter is varied, with $e^{\Delta\epsilon_{pd}/k_BT} \in 10^{-4} \times \{5, 7, 9, 11, 13, 15\}$, denoted by the symbols, +, ×, ●, ▲, ■, ▼ respectively. D: The trend of k_A as the dimensionless binding affinity of RNAP to the DnaA promoter is varied, with $e^{\Delta\epsilon_{pd}/k_BT} \in 10^{-4} \times \{5, 7, 9, 11, 13, 15\}$, using the same symbols as in C. In all the plots, the qualitative trend is the same for all the parameter values.

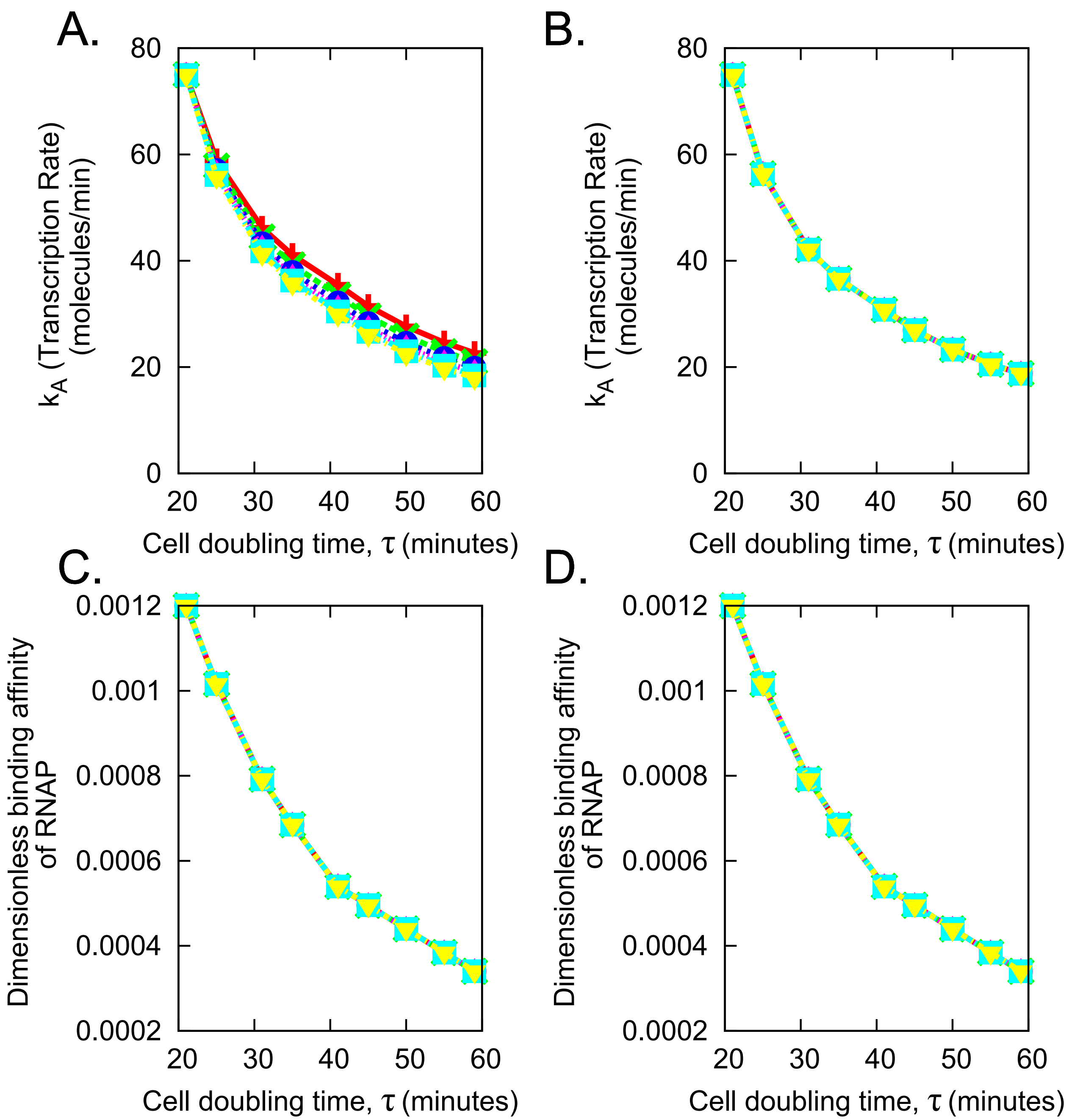

Figure A9: **The model with no autorepression but with RIDA is robust to changes in the input parameters.** A: The trend of k_A as the dimensionless binding affinity of RNAP to the DnaA promoter is varied. B: The trend of k_A as the RIDA rate, k_R is varied. C: The trend of the dimensionless binding affinity of RNAP to the DnaA promoter as k_A is varied. D: The trend of the dimensionless binding affinity of RNAP to the DnaA promoter as the RIDA rate, k_R is varied. In each case, the set different values taken by the varied parameter, and the corresponding symbols in the plots, are the same as those given in the caption of Additional Figure A8.

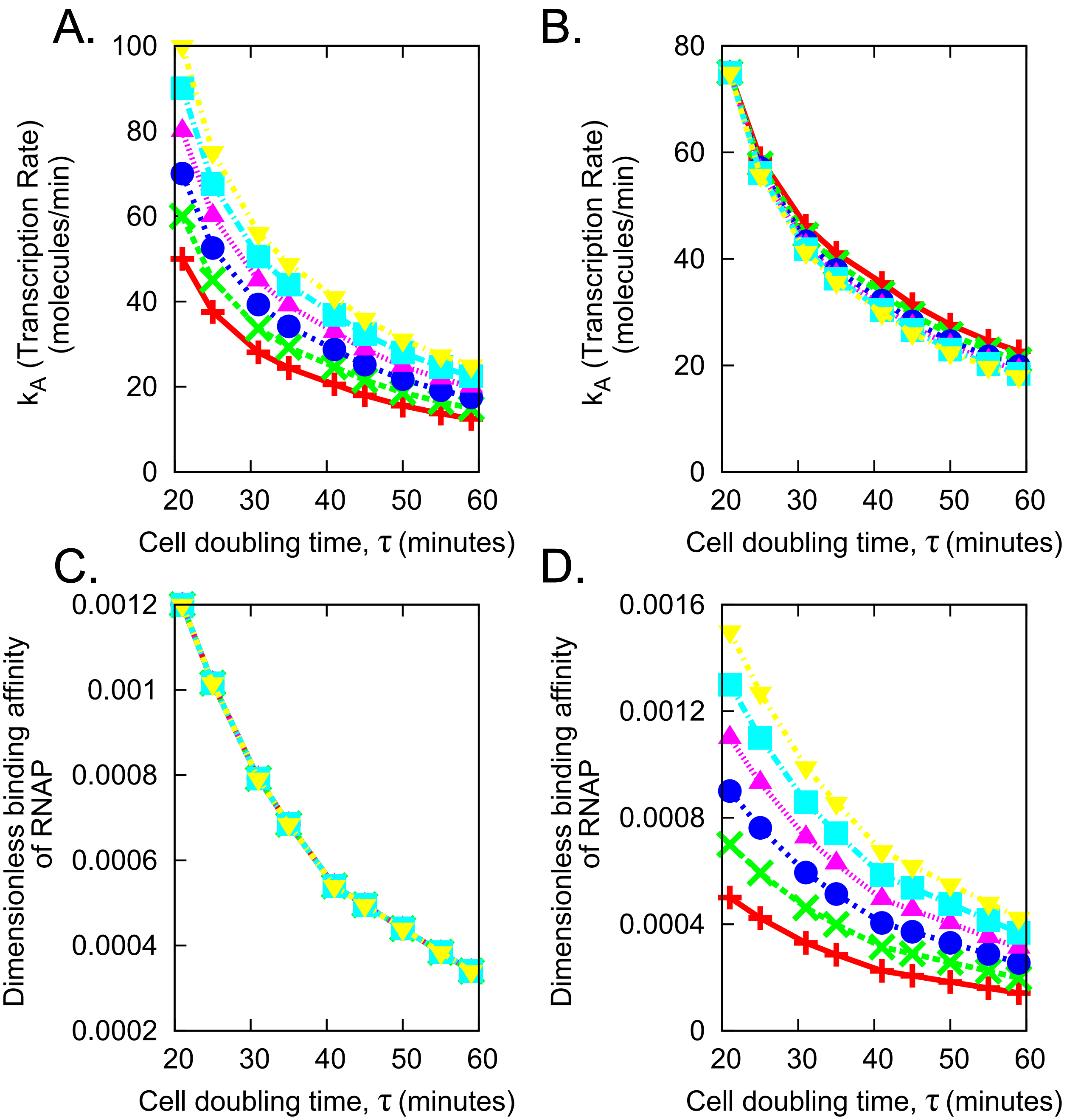

Figure A10: **The model with no autorepression and no RIDA is robust to changes in the input parameters.** A: The trend of k_A as the input value of k_A is varied. B: The trend of k_A as the dimensionless binding affinity of RNAP to the DnaA promoter is varied. C: The trend of the dimensionless binding affinity of RNAP to the DnaA promoter as k_A is varied. D: The trend of the dimensionless binding affinity of RNAP to the DnaA promoter is varied. In each case, the set different values taken by the varied parameter, and the corresponding symbols, are the same as those given in the caption of Figure A8. Note that the spread seen in A and D is due to the fact that these plots are relative to parameters whose initial values (at $\tau = 21$mins) are themselves changed. The observation that the transformation makes the initial spread at $\tau = 60$mins narrower for larger τ is further evidence of robustness.

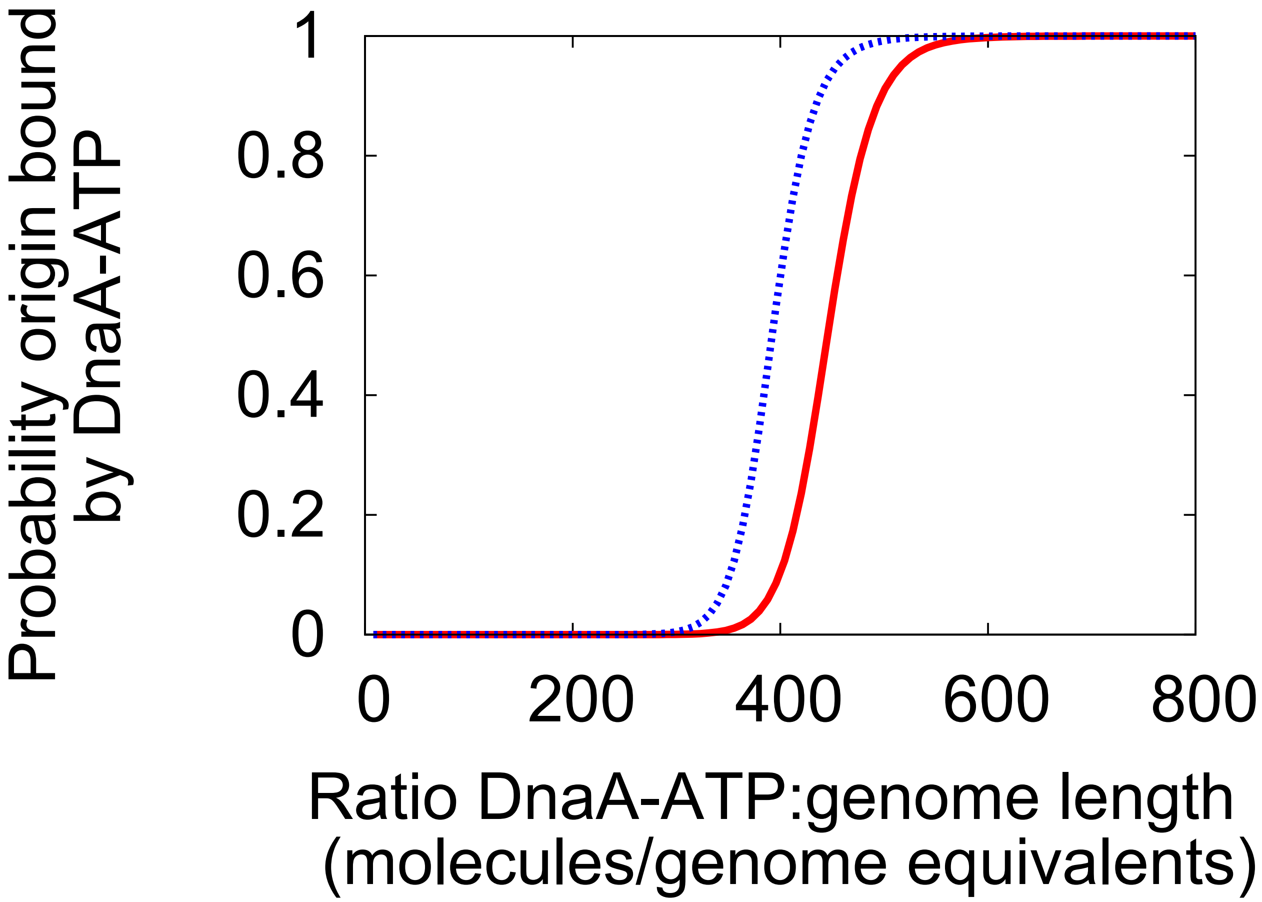

Figure A11: **Varying the binding affinity of DnaA-ATP to the sites at the origin has only a minor effect on the initiation threshold.** The probability of twenty DnaA-ATP molecules binding to the promoter, and hence starting initiation (as given in equation (26)) was plotted for different values of the binding affinity $e^{\Delta\varepsilon_{ao}/k_BT}$, to examine whether this had a large effect on the initiation threshold. The values chosen for the binding affinity were $e^{\Delta\varepsilon_{ao}/k_BT} = 0.0001$ (solid red curve), and $e^{\Delta\varepsilon_{ao}/k_BT} = 0.000088$ (dashed blue curve), which are the extreme values that the binding affinity of DnaA-ATP to its self repression sites attains in Scenario 1a (see Figure 4). The initiation threshold is given by the inflection point of the curve. Thus, the difference between the initiation thresholds is ≈ 40 (molecules/genome equivalents) $\approx 10\%$ change. This is approximately constant, particularly when compared with the differences in $r(X)$ attained in the untransformed model (Figure 3A), suggesting that this scenario might be robust.
